# SETD7-mediated monomethylation is enriched on soluble Tau in Alzheimer’s disease

**DOI:** 10.1186/s13024-021-00468-x

**Published:** 2021-07-02

**Authors:** Maria Bichmann, Nuria Prat Oriol, Ebru Ercan-Herbst, David C. Schöndorf, Borja Gomez Ramos, Vera Schwärzler, Marie Neu, Annabelle Schlüter, Xue Wang, Liang Jin, Chenqi Hu, Yu Tian, Janina S. Ried, Per Haberkant, Laura Gasparini, Dagmar E. Ehrnhoefer

**Affiliations:** 1 BioMed X Institute, Im Neuenheimer Feld 515, 69120 Heidelberg, Germany; 2 Present address: Centre for Genomic Regulation (CRG), The Barcelona Institute of Science and Technology, Dr. Aiguader 88, 08003 Barcelona, Spain; 3 AbbVie Deutschland GmbH & Co. KG, Neuroscience Discovery, Knollstr. 50, 67061 Ludwigshafen am Rhein, Germany; 4 Present address: Life Sciences Research Unit, University of Luxembourg, L-4367 Belvaux, Luxembourg; 5 Luxembourg Centre for Systems Biomedicine, University of Luxembourg, L-4362 Esch-sur-Alzette, Luxembourg; 6 AbbVie Bioresearch Center (ABC), 100 Research Dr, Worcester, MA 01605 USA; 7 AbbVie Deutschland GmbH & Co. KG, Genomics Research Center, Knollstr. 50, 67061 Ludwigshafen am Rhein, Germany; 8 European Molecular Biology Laboratory (EMBL), Meyerhofstraße 1, 69117 Heidelberg, Germany

**Keywords:** Lysine methylation, Protein methyl transferase, Nuclear tau, Posttranslational modification

## Abstract

**Background:**

Human tauopathies including Alzheimer’s disease (AD) are characterized by alterations in the post-translational modification (PTM) pattern of Tau, which parallel the formation of insoluble Tau aggregates, neuronal dysfunction and degeneration. While PTMs on aggregated Tau have been studied in detail, much less is known about the modification patterns of soluble Tau. Furthermore, PTMs other than phosphorylation have only come into focus recently and are still understudied. Soluble Tau species are likely responsible for the spreading of pathology during disease progression and are currently being investigated as targets for immunotherapies. A better understanding of their biochemical properties is thus of high importance.

**Methods:**

We used a mass spectrometry approach to characterize Tau PTMs on a detergent-soluble fraction of human AD and control brain tissue, which led to the discovery of novel lysine methylation events. We developed specific antibodies against Tau methylated at these sites and biochemically characterized methylated Tau species in extracts from human brain, the rTg4510 mouse model and in hiPSC-derived neurons.

**Results:**

Our study demonstrates that methylated Tau levels increase with Tau pathology stage in human AD samples as well as in a mouse model of Tauopathy. Methylated Tau is enriched in soluble brain extracts and is not associated with hyperphosphorylated, high molecular weight Tau species. We also show that in hiPSC-derived neurons and mouse brain, methylated Tau preferentially localizes to the cell soma and nuclear fractions and is absent from neurites. Knock down and inhibitor studies supported by proteomics data led to the identification of SETD7 as a novel lysine methyltransferase for Tau. SETD7 specifically methylates Tau at K132, an event that facilitates subsequent methylation at K130.

**Conclusions:**

Our findings indicate that methylated Tau has a specific somatic and nuclear localization, suggesting that the methylation of soluble Tau species may provide a signal for their translocation to different subcellular compartments. Since the mislocalization and depletion of Tau from axons is associated with tauopathies, our findings may shed light onto this disease-associated phenomenon.

**Supplementary Information:**

The online version contains supplementary material available at 10.1186/s13024-021-00468-x.

## Background

Tau is a predominantly cytoplasmic protein that localizes to axons in mature neurons, where it stabilizes the microtubule cytoskeleton. Mislocalization of Tau to the soma and dendrites occurs early during the development of AD and other tauopathies [1], and molecular studies have shown that acetylation at specific lysine residues can modulate this process [2]. Additionally, nuclear or perinuclear localization of Tau has been described in cultured cells as well as in human brain [3–5]. The molecular mechanism for this nuclear translocation remains unclear, since Tau does not contain a nuclear import or localization signal, and no PTM associated with nuclear Tau has been identified to date.

Tau is subject to a large variety of PTMs, with significantly altered modification patterns in AD and other neurodegenerative diseases [6–10]. In AD, Tau accumulates intraneuronally in detergent-insoluble aggregates with fibrillar morphology, termed neurofibrillary tangles (NFT). Mass-spectrometry (MS) based studies show that Tau in NFTs is heavily modified, predominantly by hyperphosphorylation and truncation [8, 10–14]. However, it has become clear that misfolded Tau species can cause significant neuronal dysfunction before the appearance of mature NFT [15, 16]. Soluble Tau isolated from human patient brains exhibits properties associated with pathology and induces the aggregation of endogenous Tau in a primary neuron model system [17]. Tau species capable of propagating Tau pathology have also been isolated from the interstitial fluid (ISF) of Tau transgenic mice, confirming their presence in the extracellular space [18, 19]. Soluble Tau species may thus be a driver in the spreading of Tau pathology throughout the brain as observed in AD. Nevertheless, the biochemical properties and PTM patterns that differentiate soluble, pathogenic Tau from physiological species remain unknown.

Here we demonstrate using an unbiased mass spectrometry (MS)-based method [20] that lysine monomethylation is a highly prevalent PTM on soluble Tau. We identified six novel methylation sites, two of which are enriched in the soluble fraction of AD brain lysate. Monomethylation at specific lysines increases with age in the brain of a mouse model of tauopathy, and is enriched in a pool of soluble Tau. We also find that the protein lysine methyltransferase (PKMT) SETD7 regulates Tau methylation at two sites in close vicinity, and these modified Tau species preferentially localize to nuclear fractions in hiPSC-derived neurons as well as in the brain of rTg4510 mice.

## Materials and methods

### Human brain samples

Anonymized human post-mortem tissue was obtained from the London Neurodegenerative Diseases Brain Bank and the Southwest Dementia Brain Bank, members of the Brains for Dementia Research Network, as well as the Netherlands Brain Bank (NBB), Netherlands Institute for Neuroscience, Amsterdam. Donor characteristics are described in Supplementary Tables S1 and S3. All Material has been collected from donors for whom a written informed consent for a brain autopsy and the use of the material and clinical information for research purposes had been obtained by the respective brain bank.

### Transgenic mouse brain samples

Heterozygous rTg4510 mice and the corresponding wild-type (WT) line (licensed from the Mayo Clinic, Jacksonville Florida, USA) [21] were used in the study. All mice were bred for AbbVie by Charles River Laboratories (Sulzfeld, Germany). The mice were in a temperature- and humidity-controlled room with a 12:12 h dark/light cycle with ad libitum access to water and food. All animal experiments were performed in full compliance with the Principles of Laboratory Animal Care (NIH publication No. 86–23, revised 1985) in an AAALAC accredited program where veterinary care and oversight was provided to ensure appropriate animal care. All animal studies were approved by the government of Rhineland Palatinate (Landesuntersuchungsamt Koblenz) and conducted in accordance with the directive 2010/63/EU of the European Parliament and of the Council on the protection of animals used for scientific purpose, the ordinance on the protection of animals used for experimental or scientific purposes (German implementation of EU directive 2010/63; BGBl. I S. 3125, 3126), the Commission Recommendation 2007/526/EC on guidelines for the accommodation and care of animals used for experimental and other scientific purposes, the German Animal Welfare Act (BGBl. I S. 1206, 1313) amended by Art. 1 G from 17 December 2018 I 2586. Tg4510 mice express 0N4R human P301L mutant hTau under the CaMKIIα promoter. For this study, mice were culled and hippocampi and cortices were harvested from 8-, 16- and 30-week old heterozygous rTg4510 mice and 16 week old WT mice and stored at − 80 °C until use.

### Brain lysate preparation

Human and mouse brain samples were homogenized in Triton lysis buffer (150 mM NaCl, 20 mM Tris pH 7.5, 1 mM EDTA, 1 mM EGTA, 1% Triton-X100 and protease, phosphatase, demethylase (500 μM IOX1, 2 μM Daminozide, 10 μM Paragyline Hydrochloride), deacetylase (10 μM Trichostatin A, 5 mM Nicotinamide), O-GlcNAcase (1 μM Thiamet-G) inhibitors) with a Dounce homogenizer (Carl Roth). The tubes were twice scraped over a hard surface with in-between incubation on ice for 10 min. Samples were centrifuged for 20 min at 16000x g at 4 °C, and the protein concentration in the supernatant was determined with a BCA assay (BioRad, cat. No. 5000112).

Triton-insoluble pellets from mouse brain were resuspended in sample buffer (65 mM Tris-HCl, 5% Glycerol, 1% SDS) and boiled for 15 min at 95 °C, followed by centrifugation for 25 min at 16000x g, 4 °C. The supernatant containing Triton-insoluble Tau was used for subsequent Western blot analyses.

### Sarkosyl extraction of human brain samples

Sarkosyl extraction was performed as described previously ( [22, 23], Suppl. Fig. S3). Briefly, brain tissue was homogenized in TBS buffer (50 mM Tris pH 7.4, 150 mM NaCl, 20 mM NaF, 1 mM Na_3_VO_4_, 0.5 mM MgSO_4_, complete protease inhibitor with EDTA (Roche), PhosSTOP phosphatase inhibitor (Roche)) with a cooled bead ruptor (FastPrep-24, MP Biomedicals) at 5 μl/mg tissue. Homogenates were centrifuged at 270000 x g and 4 °C for 20 min to obtain the supernatant S1 and pellet P1. S1 was flash frozen and stored at − 80 °C until use. P1 was suspended in the original volume of salt/sucrose buffer (10 mM Tris/HCl pH 7.4, 1 mM EGTA, 0.8 M NaCl, 10% sucrose, complete protease inhibitor with EDTA (Roche) and PhosSTOP phosphatase inhibitor (Roche)) at room temperature (RT) with a bead ruptor (Omni International, Inc). Samples were centrifuged at 27000x g and 4 °C for 20 min, the resulting supernatant S2 was adjusted to a final concentration of 1% sarkosyl and incubated for 1.5 h at RT with 750 rpm shaking. Samples were centrifuged at 150000x g and 4 °C for 45 min, the sarkosyl-insoluble pellet P3 was washed once in TBS (25 mM Tris pH 7.4, 3 mM KCl, 140 mM NaCl) and resuspended in TBS by vortexing and four sonication pulses (2 s each) with a probe sonicator (Sonoplus, Bandelin) at 35% amplitude. For ELISA, the extracts were mixed with an equal volume of 10 μl 2x Laemmli buffer (Bio-Rad), heated for 5 min at 98 °C, flash frozen and stored at − 80 °C until use.

### Immunoprecipitation of brain samples and tau PTM analysis by LC-MS/MS

250 μg protein from each human brain sample lysed in Triton lysis buffer was used for immunoprecipitation (IP) with a combination of Tau 12 (Biolegend, cat. no. 806501), Tau 5 (Abcam, cat. no. ab80579) and HT7 (Thermo Fisher, cat. no. MN1000) as described previously [20]. Briefly, immunoprecipitated samples were eluted with 50 mM glycine HCl pH 2.8, separated by SDS-PAGE and Coomassie-stained bands were excised. Samples were subjected to either an in-gel tryptic- or to an in-gel AspN digest, and peptides were separated using nanoAcquity UPLC (Waters) with a nanoAcquity trapping (nanoAcquity Symmetry C18, 5 μm, 180 μm × 20 mm) and analytical column (nanoAcquity BEH C18, 1.7 μm, 75 μm × 200 mm), which was coupled to an LTQ Orbitrap Velos Pro (Thermo Fisher) using the Proxeon nanospray source as previously described [20].

Acquired LC-MS/MS data were processed using IsobarQuant [24] and Mascot (v2.2.07) with a reversed Uniprot *Homo sapiens* database (UP000005640) including common contaminants. The following modifications were taken into account: Carbamidomethyl (C) (fixed modification), as well as Methyl (K), Dimethyl (K), Acetyl (K), Acetyl (N-term), Phospho (ST), Phospho (Y) and Oxidation (M) (variable modifications). The mass error tolerance for full scan MS spectra was set to 10 ppm and for MS/MS spectra to 0.5 Da. A maximum of 2 missed cleavages were allowed. A minimum of 2 unique peptides with a peptide length of at least seven amino acids and a false discovery rate below 0.01 were required on the peptide and protein level. Peptides with a MASCOT Score ≥ 20 were determined as reliable detected peptides [25]. The data have been deposited to the ProteomeXchange Consortium via the PRIDE [26] partner repository with the dataset identifier PXD017065. 

### Methyl- tau antibodies

Immunization and antibody purification were performed by Innovagen (Lund, Sweden). Antibodies were raised in rabbits against the respective methylated peptides indicated in Suppl. Fig. S1. The animals were subjected to a total of three booster injections, with the final immune serum harvest 2 months after the first immunization. Immune sera were affinity purified against the appropriate methyl-peptide, and the purified antibodies were stored in aliquots in PBS at − 80 °C until use.

### Recombinant tau purification

Full length Tau 2N4R was expressed and purified as described previously [20]. Briefly, *E. coli* BL21(DE3) cells (Sigma, cat. no. CMC0014) transformed with a pET19n vector containing 2N4R Tau were induced by the addition of 1 mM IPTG and harvested by centrifugation. Cleared lysates in 50 mM Na-phosphate pH 7.0, 1 mM EGTA, 1 mM DTT, cOmplete protease inhibitors (Roche), benzonase (Merck) and 10 μg/ml lysozyme (Sigma) were boiled for 20 min at 100 °C. Supernatants were loaded onto a combination of a HiTrap Q and a HiTrap SP column (GE Healthcare) and eluted in a gradient to running buffer containing 300 mM Nac*l. tau*-containing fractions were further purified with a HiLoad 16/600 Superdex 75 pg size exclusion chromatography column (GE Healthcare). Fractions were analyzed by SDS-PAGE, pooled according to purity, flash-frozen in liquid nitrogen and stored at − 80 °C.

### In vitro methylation of recombinant tau

100 μg of recombinant Tau 2N4R were incubated for 2 h at RT in 100 mM sodium citrate pH 6 (Sigma, cat. no. W302600), 100 mM sodium cyanoborohydride (Sigma, cat. no. 156159) and 20 mM formaldehyde (Fisher Scientific, cat. no. 28908) with a final volume of 100 μl. 10 μl of 550 mM glycine (Roth, cat. no. 3187) were added, and Zeba™ Spin desalting columns (Thermo Fisher, cat. no. 87767) were used according to manufacturer’s instructions to remove excessive sodium cyanoborohydride.

### In vitro dephosphorylation with lambda phosphatase

5 μl aliquots of the respective brain fractions were mixed with 1 μl lambda phosphatase (New England Biolabs, cat. no. P0753), 10x buffer and MnCl2 were added according to manufacturer’s instructions in a total volume of 10 μl. Control reactions were set up without addition of the enzyme. After incubation for 30 min at 30 °C, 2.5 μl of 4x Laemmli sample buffer were added, and samples were processed as described below for immunoblotting.

### Immunoblotting

To generate dot blots for testing the specificity of methyl-Tau antibodies, 3 μg methylated peptides, bare peptides, in vitro methylated full-length Tau (2N4R) and recombinant Tau (2N4R) were spotted onto nitrocellulose membranes.

For Western blots, 25 μg total protein were boiled at 95 °C for 5 min in sample buffer, separated on a 10% SDS-PAGE and immunoblotted onto PVDF membranes.

The membranes were blocked with blocking buffer (5% BSA in TBST (Roth)) and incubated with primary antibodies overnight at 4 °C: Tau 12, mouse, 1:500, (Biolegend, cat. no. 806501); Tau 5, mouse, 1:1000, (Abcam, cat. no. ab80579); meK130, rabbit, 1:500, (INNOVAGEN); meK132, rabbit, 1:1000 (INNOVAGEN); meK343, rabbit, 1:1000 (INNOVAGEN); meK353, 1:200, (INNOVAGEN); meK438, rabbit, 1:1000 (INNOVAGEN); Actin, rabbit 1:1000, (Cell Signaling Technology, cat. no. 4910); SETD7, mouse, 1:1000, (Thermo Fisher, cat. no. 730055); SETD7, rabbit, 1:1000, (Invitrogen, cat. no. MA5–35782); Hsp90, mouse, 1:1000, (Millipore, cat. no. 05–594); HDAC2, rabbit, 1:1000, (Cell Signaling Technology, cat. no. 2545S). Antibodies were diluted in 5% BSA in 1X TBST (1X TBS, 0.05% Tween-20). The next day, secondary antibody incubations (1:20000, IRDye Donkey anti-mouse 800 and IRDye Donkey anti-rabbit 680) were performed in blocking solution for 1 hour at RT. The membranes were then washed three times with 1X TBST, once with 1X TBS and imaged on a Li-Cor Odyssey CLx scanner. Alternatively, AzureSpectra antibodies (IRDye goat anti-mouse 800, cat. no. 201206–58, 1:5000, and IRDye goat anti-rabbit 650, cat. no. 180309–72,1:5000) were used and imaged using ChemiDoc Touch MP (Bio-Rad) and analysed via ImageLab6.

For weak signals, anti-rabbit HRP- linked antibody was used (Cell Signaling Technology, 1:5000) in blocking buffer, with incubation for 1 hour at RT. The membranes were then washed three times with 1X TBST, once with 1X TBS. ECL solution clarity Max (Bio-Rad, cat. no. 1705062) was added to the membrane and chemiluminescent signals were detected on a Fusion FX7 (Vilber).

### Electrochemiluminescence ELISA

To quantify methylated Tau in mouse brain samples and SHSY5Y cells, Gold Streptavidin small-spot 96-well plates (Meso Scale Discovery, cat. no. L45SA) were blocked with 5% (w/v) Blocker A solution in Tris wash buffer for 1 h at RT on a plate shaker. Plates were washed three times with Tris wash buffer and coated with 1 μg of biotinylated antibody diluted in 25 μL of 1% Blocker A solution for 1 h at RT on a plate shaker. Antibody biotinylation was performed according to manufacturer’s instructions (EZ-Link Sulfo-NHS-Biotin, Thermo Scientific, cat. no. 21217), after BSA removal with the Melon Gel IgG Purification Kit (Thermo Scientific, cat. no 45212) if necessary. For mouse brain samples, biotin Tau5 (Abcam) was used as a total Tau antibody. For SHYSY5Y cells, biotin HT7 (Thermo Fisher, cat. no. MN1000) was used. Antibody-coated plates were washed three times with Tris wash buffer, 5 μg of protein lysates (diluted to 50 μl with TBS) were added and incubated for 1 h at RT on a plate shaker. Plates were washed three times with Tris wash buffer and incubated for 1 h at RT on a plate shaker with 25 μl of 1 μg/ml detection antibody (for mouse samples: HT7; for SHSY5Y samples: Tau 12, both labeled with MSD Sulfo-Tag-NHS-Ester, Meso Scale Discovery) diluted in 1% Blocker A solution. Plates were washed three times with Tris wash buffer and 150 μl of 2X Read Buffer were added 5 minutes before the signal was measured on a MESO QuickPlex SQ 120 (Meso Scale Discovery).

### Colorimetric ELISA

ELISA plates (Maxi Sorp, Thermo Scientific) were coated over night at 4 °C with 100 μl capture antibody per well (2 μg/ml Tau-12 (Biolegend, cat. no 806502), meK130 or meK132, AT8 (Invitrogen, cat. no. MN1020) or AT100 (Invitrogen, cat. no. MN1060) antibody in 20 mM NaH_2_PO_4_ pH 7.4, 140 mM NaCl, 20% glycerol). The next day, plates were washed 3x with 250 μl/well wash buffer (20 mM NaH_2_PO_4_ pH 7.4, 140 mM NaCl, 0.05% Tween 20) and blocked for 1.5 h at RT with 250 μl/well blocking buffer (20 mM NaH_2_PO_4_ pH 7.4, 140 mM NaCl, 0.05% Tween 20, 20% glycerol, 2% BSA). Samples were diluted in assay buffer (20 mM NaH_2_PO_4_ pH 7.4, 140 mM NaCl, 0.05% Tween 20, 0.1% BSA) to fall within the experimentally validated linear range of the assay. Plates were washed 3x with 250 μl/well wash buffer and 100 μl/well samples were added. All incubation steps of meK132 and meK130 ELISA plates were performed on an orbital shaker at 650 rpm. After 2 h incubation at RT, plates were washed 3x with 250 μl/well wash buffer. For plates with Tau 12 as capture antibody, 100 μl/well of 0.1 mg/ml Biotin-HT7 antibody (Invitrogen, MN1000B) in assay buffer were added. For plates with meK130, meK132, AT8 or AT100 as capture antibodies, 100 μl/well of 0.1 mg/ml Biotin-Tau 12 (Biolegend) were added. Plates were incubated for 1 h at RT, washed 3x with 250 μl/well wash buffer, and 100 μl/well of Pierce Streptavidin poly-HRP conjugate were added (Thermo Scientific, diluted 1:10000 in assay buffer). The plates were incubated for 1 h at RT, washed 3x with 250 μl/well wash buffer, and 100 μl/well TMB ELISA substrate were added (KEM EN TEC Diagnostics). After 10 min incubation at RT in the dark, the reaction was stopped by adding 100 μl/well 0.18 M H_2_SO_4_, and the absorbance was read at 450 nm.

### Culture and transfection of cell lines

HEK 293 T cells were cultured at 37 °C in 5% CO_2_ in DMEM+ GlutaMax (Thermo Fisher, cat. no. 10566016) with 10% fetal bovine serum (Sigma, cat. no. F7524-500 ml) and 1% penicillin/streptomycin (Fisher Scientific, cat. no. 15307583). Cells were used for transfections between passage 10–20. Transfection was performed at a cell confluency of 60–70% using JetPrime reagent (PolyPlus) according to manufacturer’s instructions. For each construct, 1 μg DNA (WT 2N4R Tau, K130R 2N4R Tau, K132R 2N4R Tau, K343R 2N4R Tau, K353R 2N4R Tau and K438R 2N4R Tau) was used. Tau constructs contained an N-terminal HA tag and were cloned into the pcDNA3 vector, mutants were generated by site-directed mutagenesis (Q5 site directed mutagenesis, New England BioLabs, cat. no. E0552S) and verified by sequencing. Cells were transfected and incubated for 24 h before they were harvested by scraping, centrifuged for 5 min at 2000x g at 4 °C, washed once with ice-cold PBS and centrifuged again for 5 min at 2000x g at 4 °C. Samples were stored at − 20 °C until further use.

SHSY5Y neuroblastoma cells stably expressing GFP-0N4R Tau (Innoprot, cat. no. P30722-4R) were cultured at 37 °C in 5% CO_2_ in RPMI 1640 + GlutaMax media (Thermo Fisher, cat. no. 61870044) with 10% fetal bovine serum and 250 μg/G418 (Fisher Scientific, cat. no. 10131035). Cells were used for experiments between passage 3–20. 5 μM (R)-PFI-2 (Sigma cat. no. SML1408) was added to the cells at a confluency of 50%, followed by an incubation for 16 h. Cells were harvested by scraping, centrifuged for 5 min at 2000x g at 4 °C, washed once with ice-cold PBS and centrifuged for 5 min at 2000x g at 4 °C. Samples were stored at − 20 °C until further use.

### Treatment with PKMT inhibitors and cell viability test

Compounds described as selective PKMT inhibitors by the Structural Genomics Consortium (SGC, [27]) were sourced as outlined in Suppl. Table S2, stock solutions were prepared in DMSO and stored at − 20 °C until use. SHSY5Y cells were seeded into 96well plates and treated with PKMT inhibitors in the medium at the concentrations indicated in Suppl. Table S2 for 24 h. Cell titer blue reagent (Promega) was added according to manufacturer’s instructions, incubated with the cells for 3 h and fluorescence was measured with an excitation at 560 nm and emission at 590 nm. Cells were washed once with PBS, lysed and processed for ELISA as described above.

### hiPSC neuron culture

Neurogenin 2 (NGN2)- induced neurons were differentiated as previously described with minor modifications [28]. A doxycycline inducible NGN2 expression cassette was stable integrated in the AAV1 locus using TALEN technology by Bioneer (Denmark). hiPSCs were split in a concentration of 100,000 cells/cm^2^ on matrigel (BD) coated plates in mTesR (Stem Cell Technologies). At day 1 after splitting, the medium was changed to N2/B27 medium (50% DMEM/F12, 50% Neurobasal, 1:200 N2, 1:100 B27, 1% PenStrep, 0.5 mM Non-essential amino acids, (all Invitrogen), 50 μM ß-mercaptoethanol (Gibco), 2.5 μg/ml insulin and 1 mM sodium pyruvate (both Sigma)) with 2 μg/ml doxycycline (Sigma). The medium was changed daily. On day 4, cells were split with accutase (Invitrogen) and re-seeded in a density of 200,000 cells/cm^2^ in N2/B27 medium with doxycycline and 10 μM Rock inhibitor Y- 27632 (Selleckchem) on matrigel coated plates in the final format. N2/B27 with doxycycline was changed daily until day 7. On day 8 the medium was switched to final maturation medium (FMM; N2/B27 with 20 ng/ml BDNF, 10 ng/ml GDNF (both Peprotech), 1 mM dibutyryl-cAMP (Sigma) and 200 μM ascorbic acid (Sigma)). The medium was changed every third day until cells were used for treatment with (R)PFI-2 at day 21. N2/B27 medium with DMSO or 5 μM (R)-PFI-2 [29] were added and refreshed once after 24 h. 48 h after initial treatment, cells were detached with ice-cold PBS and centrifuged for 5 min at 2000x g at 4 °C. Samples were stored at − 20 °C until further use.

### Proximity ligation assay (PLA)

Specific primary antibodies against Tau (Tau12, mouse, 1:500, Biolegend, cat. no. 806501) and total mono-methyl Lysine (meK, rabbit, 1:500, Cell Signaling, cat. no. 14679S) were used to detect methylated Tau in hiPSC-derived neurons. A combination of the Tau12 antibody with an additional total Tau antibody (Tau, rabbit, 1:500, Dako, cat. no. A0024) served as positive control. Negative controls consisted of applying only one primary antibody against Tau (Tau12) and of omitting the primary antibodies (not shown). PLA was performed in MAPT wildtype and MAPT knockout hiPSC-derived neurons. Duolink In Situ PLA red mouse/rabbit kit was purchased from Sigma Aldrich (DUO92002, DUO92004, DUO82049, DUO92008; Sigma-Aldrich, St. Louis, Missouri, USA). This kit includes mouse and rabbit secondary antibodies with probes, blocking solution, wash buffers A and B, amplification solution, ligase solution and detection reagent. PLA was performed according to manufacturer’s instructions.

For PLA analysis, images were acquired using a Leica LSM800 confocal laser-scanning microscope with a 25× oil immersion objective (Leica, Wetzlar, Germany). Thickness of single optical sections was 0.83 μm in stacks of ~ 14 μm total depth. Individual images were composed of 6 tiles (3 × 2 tiles in x- and y-direction, respectively).

For quantification of methylated Tau in hiPSC-derived neurons, 9 images per condition were analyzed for each biological replicate. Three technical and biological replicates for each condition were applied. Nuclear meK-Tau12 PLA signal in hiPSC-derived neurons was measured using ImageJ (Fiji). The integrated density of PLA probe signal in nuclei was quantified in a mask created of Hoechst-labeled nuclei.

### SETD 7 Knock down

shRNA sequences against SETD7 were designed using an online tool (www.biosetta.com) and synthesized (Eurofins). Oligonucleotide sequences were as follows:

shRNA-1:

top: 5′ – ACCGGGCCAGGGAGTTTACACTTAGTTAATATTCATAGCTAAGTGTAAACTCCCTGGCTTTT – 3′.

bottom: 5′ – CGAAAAAAGCCAGGGAGTTTACACTTAGCTATGAATATTAACTAAGTGTAAACTCCCTGGCC – 3′.

shRNA-2:

top: 5′ – ACCGGGGGAGTTTACACTTACGAAGTTAATATTCATAGCTTCGTAAGTGTAAACTCCCTTTT – 3′.

bottom: 5′ – CGAAAAAAGGGAGTTTACACTTACGAAGCTATGAATATTAACTTCGTAAGTGTAAACTCCCC – 3′.

shRNA oligonucleotides were cloned into the DECIPHER pRSI12-U6-(sh)-HTS4-UbiC-TagRFP-2A-Puro lentiviral expression vector (Cellecta, cat. no. DVLIB-PS) following manufacturer’s instructions and then transformed into OneShot Top10 *E.coli* (Thermo Fisher, cat. no. C404003). Positives clones were verified by sequencing. 1 μg of two different shRNAs were packaged with lentiviral plasmids (MD2G, psPAX2) and transfected into HEK cells using JetPrime reagent (PolyPlus) according to manufacturer’s instructions. Media was collected 48 h after transfection and concentrated using Lenti-X™ concentrator (Takara Bio Europe SAS, cat. no. 631231) according to manufacturer’s instructions. Viral pellets were resuspended in 300 μl of DMEM and stored at − 80 °C.

SHSY5Y cells were seeded at 20% confluency in a 12 well plate before adding 10 μL lentivirus with shRNA. Cells were cultivated for 7 days with a media change on every third day. Cells were harvested by scraping, centrifuged for 5 min at 2000x g at 4 °C, washed once with ice-cold PBS and re-centrifuged for 5 min at 2000x g at 4 °C. Samples were stored at -80 °C until further use.

The knock down efficiency was analyzed using qPCR: RNA was extracted from the cell pellets using RNeasy Plus Mini Kit (QIAGEN, cat. no. 74134) according to the manufacturer’s protocol. A reverse transcription reaction was carried out to synthesize 250 ng cDNA using the High-Capacity cDNA Reverse Transcription Kit (Fisher Scientific, cat. no. 10400745), and qPCR was performed with the GoTaq® qPCR Master Mix (Promega, cat. no. A6001) following the manufacturer’s protocol on a Quant Studio 3 real-time PCR machine (Thermo Fisher).

### Subcellular fractionation of mouse brain and cultured neurons

For subcellular fractionation, hiPSC-derived neurons were washed off the culture plate in ice-cold PBS, pelleted and fractionated using the Pierce Subcellular fractionation kit for cultured cells (Thermo Fisher) according to manufacturer’s instructions. Mouse brain samples were fractionated using the Pierce Subcellular fractionation kit for tissues (Thermo Fisher) according to manufacturer’s instructions. For analysis, 2–10 μl of each fraction were run on an SDS-PAGE and subjected to Western blotting as described above.

### MS proteomics analysis of subcellular fractions

Each fraction sample (100 μl) was reduced by 1 μl 1 M Dithiothreitol (DTT) at 60 °C for 30 min and alkylated by 4 μl 1 M iodoacetamide (IAM) in the darkroom for 30 min. Then proteins were precipitated with 7 volumes of chilled acetone at − 20 °C overnight. Proteins were then pelleted by centrifugation at 14,000x g for 10 min at 4 °C. Supernatant was carefully removed, and pellets were rinsed with 500 μl methanol. Protein pellets were resuspended in 80 μL of 100 mM ammonium bicarbonate and digested by addition of trypsin/Lys-C (Promega, Madison, WI) at a protein-to-total enzyme ratio of 50:1 at overnight. Trifluoroacetic acid (TFA) was added to a final concentration of 1% (v/v) to quench the digestion. Peptide concentration was determined by measuring optical density (OD) at 280 nm using Trinean DropSense 96 (PerkinElmer, Waltham, MA). Peptide concentration was then adjusted to 1 μg/μl using 2% methanol, 0.1% TFA in water and transferred to sample vial for LC-MS/MS analysis.

Peptide samples were analyzed using a Thermo Scientific EASY-nLC 1000 system coupled to a Orbitrap Fusion Lumos Mass Spectrometer (Thermo Scientific, Waltham, MA). The LC system was equipped with a PepMapTM RSLC C18 column (75 μm × 75 cm, 2 μm, Thermo Scientific, Waltham, MA) maintained at 50 °C. Solvent A consisted of 0.1% formic acid in water, and solvent B consisted of 0.1% formic acid in 90% acetonitrile. A 2 μg peptide sample was loaded onto trap with 5% acetonitrile at 10 μL/min flow rate for 5 min and then analyzed at 200 /min with LC separation gradient: 5–15% B for 5 min, 15–30% B for 170 min, 30–65% for 20 min, 65–95% B for 11 min. Data collection was operated in a 3-s cycle using the data-dependent top-speed mode. The MS1 survey scan (m/z 400–1500) was at a resolution of 240,000 (FWHM@m/z = 200), with automated gain control (AGC) target of 400,000 and a maximum injection time of 50 ms. Precursors were fragmented in HCD activation mode at a normalized collision energy of 30% and the dynamic exclusion was set with 45 s. Precursors were filtered by quadrupole using an isolation window of 1.2 amu. The MS2 spectra were collected at a resolution of 15,000 in the Orbitrap, with an AGC target of 50,000 and a maximum injection time of 50 ms.

For data processing, raw files were searched against the Uniprot-SwissProt *Homo sapiens* protein database containing 20,244 entries with Proteome Discoverer version 2.0 (released in July, 2015) for protein identification. The search engine Sequest HT was used and the search parameters were set to 20 ppm tolerance for precursor ion mass and 0.02 Da for fragment ion mass. Two missed cleavages were permitted for fully tryptic peptides. Carbamidomethylation of cysteine was set as a static modification, and a dynamic modification was defined as oxidation at methionine and acetylation at the N-terminal. Target FDR (strict) for decoy database search was set to 0.01 in Percolator.

Proteins and the number of unique peptides identified in each fraction are listed in Supplementary Table S6. Proteins annotated as PKMTs by the HUGO gene nomenclature committee (HGNC, www.genenames.org) were identified in each fraction and Venn diagrams were generated using the venn.diagram function in R.

### Data analysis

Immunoblotting data were analyzed using Image Studio Lite (Li-cor Biosciences) and statistical analysis was performed with GraphPad Prism 7 (GraphPad Software) using the test noted within the respective figure legend. Full blots used for quantification are shown in Suppl. File S12.

### Bioinformatic analysis of public RNASeq data sets

The details of the Mount Sinai Brain Bank study (MSBB) and Mayo study sample collection, RNA extraction, library preparation and sequencing were described in previous publications [30, 31]. For the analysis presented here, the reprocessed and realigned counts as described in a previous publication [32] were used. In brief, FASTQ files were aligned to GENCODE24 (GRCh38) using STAR [33]. The counts for Mayo (syn8690904, syn8690799) and MSBB (syn8691099) were downloaded from the AMP-AD knowledge portal (https://adknowledgeportal.org). For Mayo, 33 samples were excluded as described in [33] and 523 samples of two brain regions were used for the analysis. For MSBB 753 samples of 4 brain regions were used for the analysis. Genes with 1 CPM in fewer than 10 samples were removed. In both studies, differential gene expression was calculated with Limma voom [34] per brain region adjusted for RNA integrity value (RIN), age at death, post-mortem interval (PMI) and gender, in addition mayo for study center and flow cell and MSBB for race. For Mayo, the group comparisons for AD/controls/pathological aging and Braak stage V-VI/Braak stage III-IV/Braak stage 0-II were calculated, and pair-wise contrasts extracted. For MSBB the outcome groups AD/controls, Braak stage V-VI/Braak stage III-IV/Braak stage 0-II and demented (CDR 3–5)/MCI (CDR 1–2)/non dementia (CDR 0–0.5) were analyzed. Quality control, differential gene expression and plotting was performed in R [35].

## Results

### Monomethylation of soluble tau is abundant in human brain

**Fig. 1:**
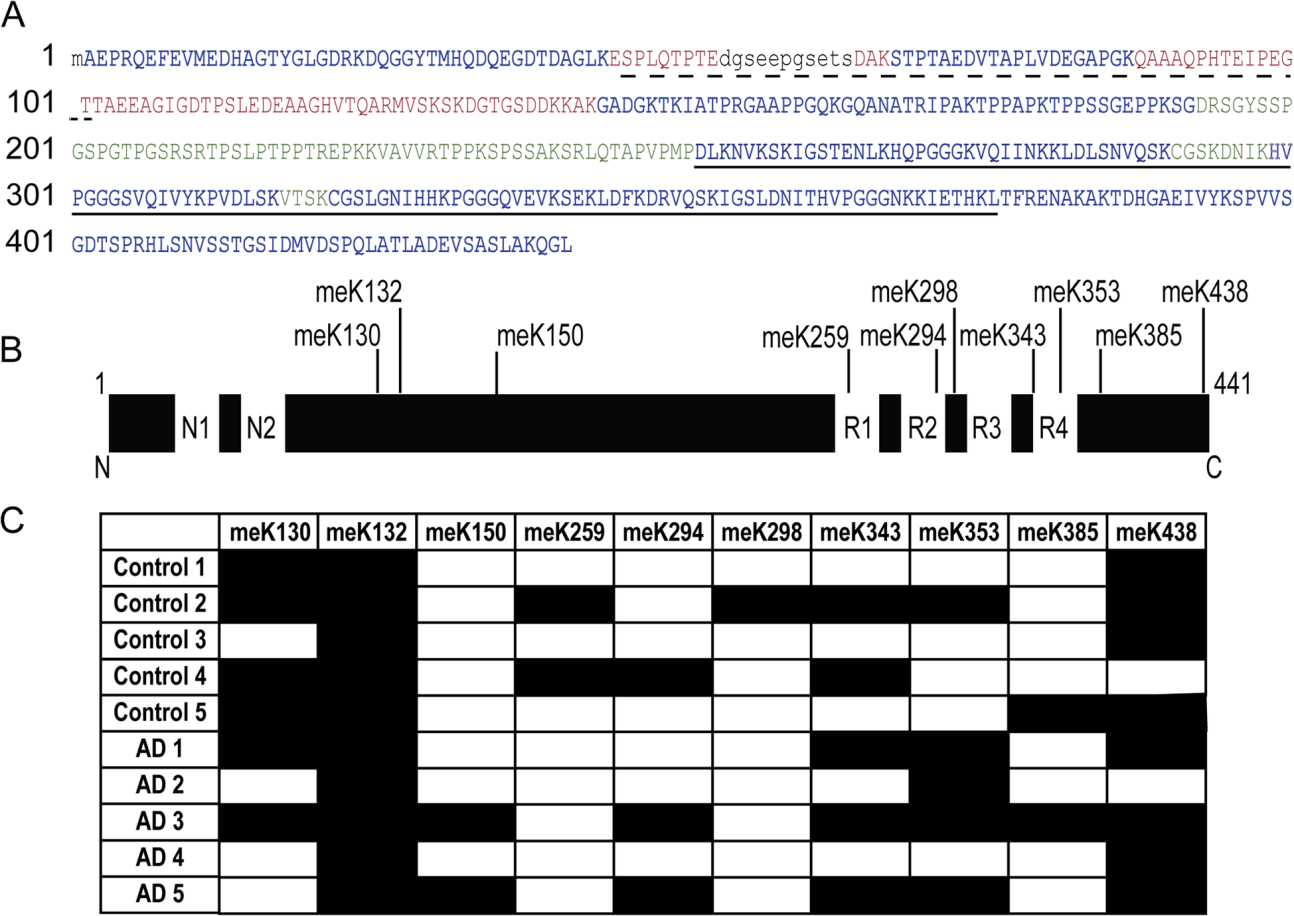
Multiple sites of lysine monomethylation detected on soluble Tau from human entorhinal cortex tissue. **A** Sequence coverage of the human 2N4R Tau isoform (UniProtKB-P10636–8). Dashed line: N-terminal repeats N1 and N2, solid line: microtubule binding domain (MTBR). Font color in blue: sequence covered by peptides obtained by both AspN and trypsin digestion, red: sequence covered exclusively by AspN-derived peptides, green: sequence covered exclusively by trypsin-derived peptides, black lower case: sequence not recovered by MS. **B** Summary of lysine monomethylation sites identified by LC-MS/MS on 2N4R Tau. **C** Prevalence of each monomethyl-modified lysine across the MS data set, black: found, white: not found

We recently developed a method to enrich detergent-soluble Tau from human brain for MS-based analyses of Tau [20]. To gain a better understanding of PTMs present at early disease stages in this soluble fraction, we analyzed entorhinal cortex samples from donors classified as Braak stages 0-I as well as Braak stages III-IV to represent non-affected controls and early AD, respectively (Suppl. Table S1). With a sequence coverage of 80–97% (Fig. 1a), we confirmed the presence of 12 phosphorylation sites and 4 sites for lysine monomethylation, which have been previously described in samples containing insoluble Tau (Suppl. Table S2, [12, 36]). In addition, we identified 6 novel lysine monomethylation sites (K130, K150, K294, K298, K343, K438) and one novel acetylation site (Suppl. Table S2). Three methylated lysines are located in the repeat domains (Fig. 1b), one in the C-terminus (meK438) and two sites in the projection domain (meK130 and meK132) and were consistently modified in multiple brain samples.

To analyze the newly identified Tau PTMs in more detail, we raised antibodies against the five most prevalent monomethyl sites, which were identified in a total of 5 or more individual brain samples: meK130, meK132, meK343, meK353 and meK438 (Fig. 1b and c). In order to verify that the novel antibodies are specific for methylated Tau, we performed dot blot assays using recombinant Tau peptides spanning the different modification sites with or without a mono-methyl modification. In addition, we used recombinant full-length Tau protein (2N4R isoform) subjected to reductive methylation, which chemically modifies all available lysine residues with methyl groups [12]. All antibodies were highly specific for the respective methylated peptides against which they were raised and recognized chemically methylated full-length Tau (Suppl. Fig. S1). To test for site specificity on cellular full-length Tau, we used transfections in HEK cells of either wild-type (WT) Tau (2N4R) or mutants at the different methyl sites (Suppl. Fig. S2). Both the meK343 and the meK438 Tau antibodies also detected the mutant protein on the Western blot and thus appeared to be non-specific with respect to their methylation site, while the specificity of meK130, meK132 and meK353 Tau antibodies was confirmed (Suppl. Fig. S2).

### meK130 and meK132-modified tau accumulates in the soluble fraction of AD brain and increases with age in rTg4510 mice

**Fig. 2:**
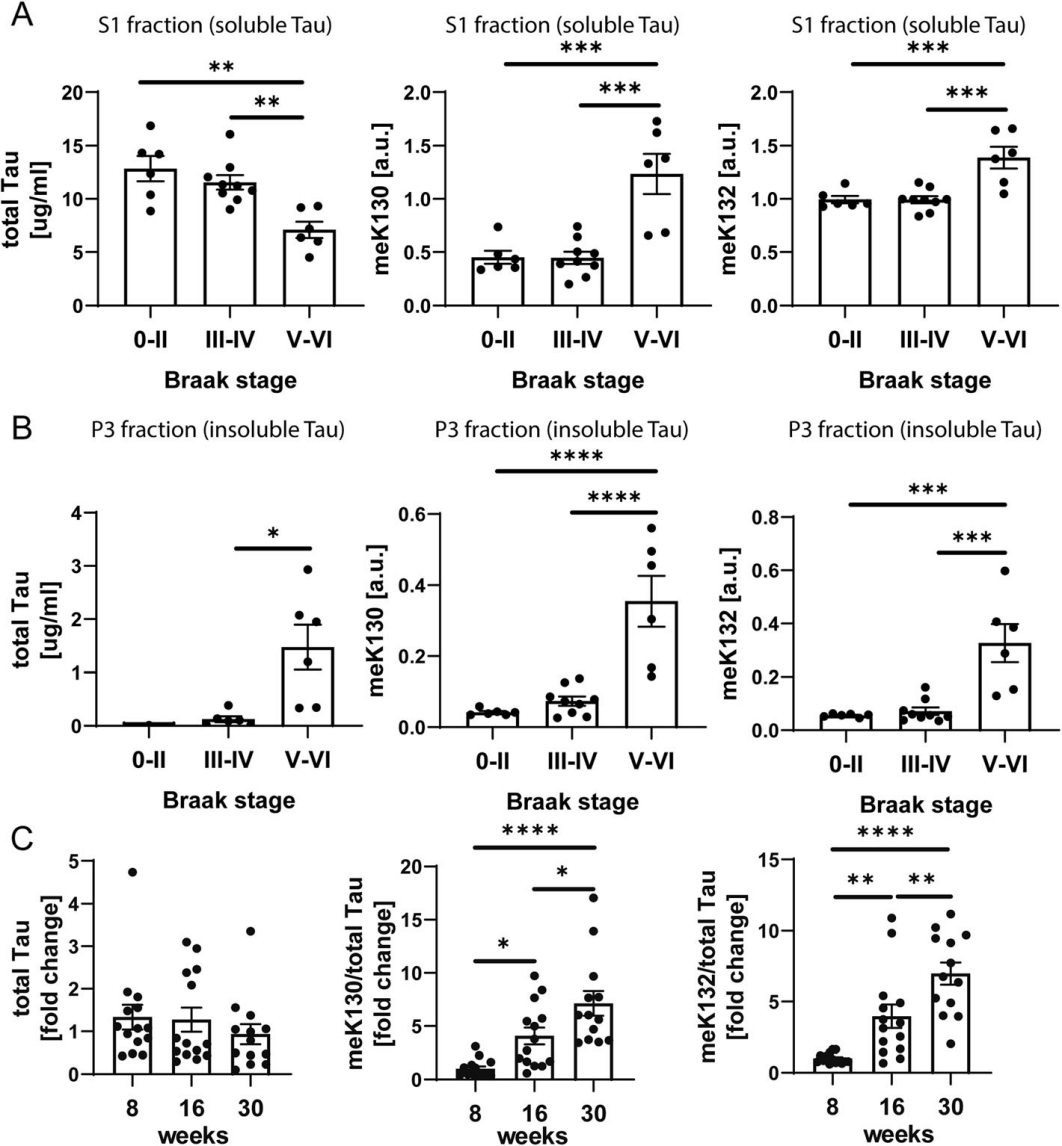
Levels of meK130 and meK132-modified Tau increase with Braak stage in humans and with age in rTg4510 mice in the soluble fraction. **A** Total Tau levels decrease while methylated Tau levels increase with Braak stage in the soluble fraction (S1) of human brain. **B** Total and methylated Tau levels increase in parallel at high Braak stages in the sarkosyl-insoluble fraction (P3). **C** Electrochemiluminescence ELISA demonstrates an age-dependent increase in meK130 and meK132-modified Tau in Triton-X soluble fractions of rTg4510 mouse hippocampus. Data are normalized to total Tau levels obtained by Tau 5 ELISA. **A**-**C** Statistical significance was determined by one-way ANOVA (S1 total Tau: ANOVA *p* < 0.001, S1 meK130: ANOVA *p* < 0.0001, S1 meK132: ANOVA *p* < 0.001; P3 total Tau: ANOVA *p* < 0.05, P3 meK130: ANOVA p < 0.0001, P3 meK132: ANOVA *p* < 0.001, rTg4510 meK130: ANOVA *p* = 0.0002, rTg4510 meK132: ANOVA *p* < 0.0001). Tukey’s multiple comparisons test was used for post-hoc analysis. *: *p* < 0.05, **: *p* < 0.01, ***: *p* < 0.001, ****: *p* < 0.0001

Mature Tau aggregates can be biochemically isolated from human brain homogenates with a step-wise centrifugation protocol using the detergent sarkosyl [22]. To determine the solubility of methylated Tau in human brain and its abundance at different disease stages, we extracted soluble and sarkosyl insoluble fractions (S1 and P3, respectively, Suppl. Fig. S3) from a set of brains spanning the whole spectrum of Braak stages (0-VI, Suppl. Table S3). We then analyzed the prevalence of meK-modified Tau in these samples by ELISA using our site-specific meK-Tau antibodies. Since antibody signal for meK353-modified Tau was very low (data not shown), we focused our efforts on the methylation sites at K130 and K132. Both meK130- and meK132-modified Tau were significantly increased in the soluble fraction Braak stages V-VI, while total Tau levels decreased in these samples (Fig. 2a). This suggests that an increasing proportion of soluble Tau species carry meK130 and meK132 modifications in late AD (Fig. 2a). In the sarkosyl-insoluble pellet, on the other hand, the abundance of meK130 and meK132-modified Tau increases proportionally with total Tau levels, suggesting that methylated Tau is not preferentially accumulating in insoluble aggregates (Fig. 2b).

Next, we investigated whether these changes are recapitulated in a transgenic mouse model of tauopathy, the rTg4510 mice [37]. rTg4510 mice progressively accumulate insoluble Tau and exhibit intraneuronal Tau inclusions throughout the forebrain from 4 months of age [21]. In agreement with our findings from human brain, soluble hippocampal fractions from mice aged 8–30 weeks showed an age-dependent increase in meK130- and meK132-modified Tau signals measured by ELISA (Fig. 2c).

### Methylation is more prevalent on nuclear tau

**Fig. 3:**
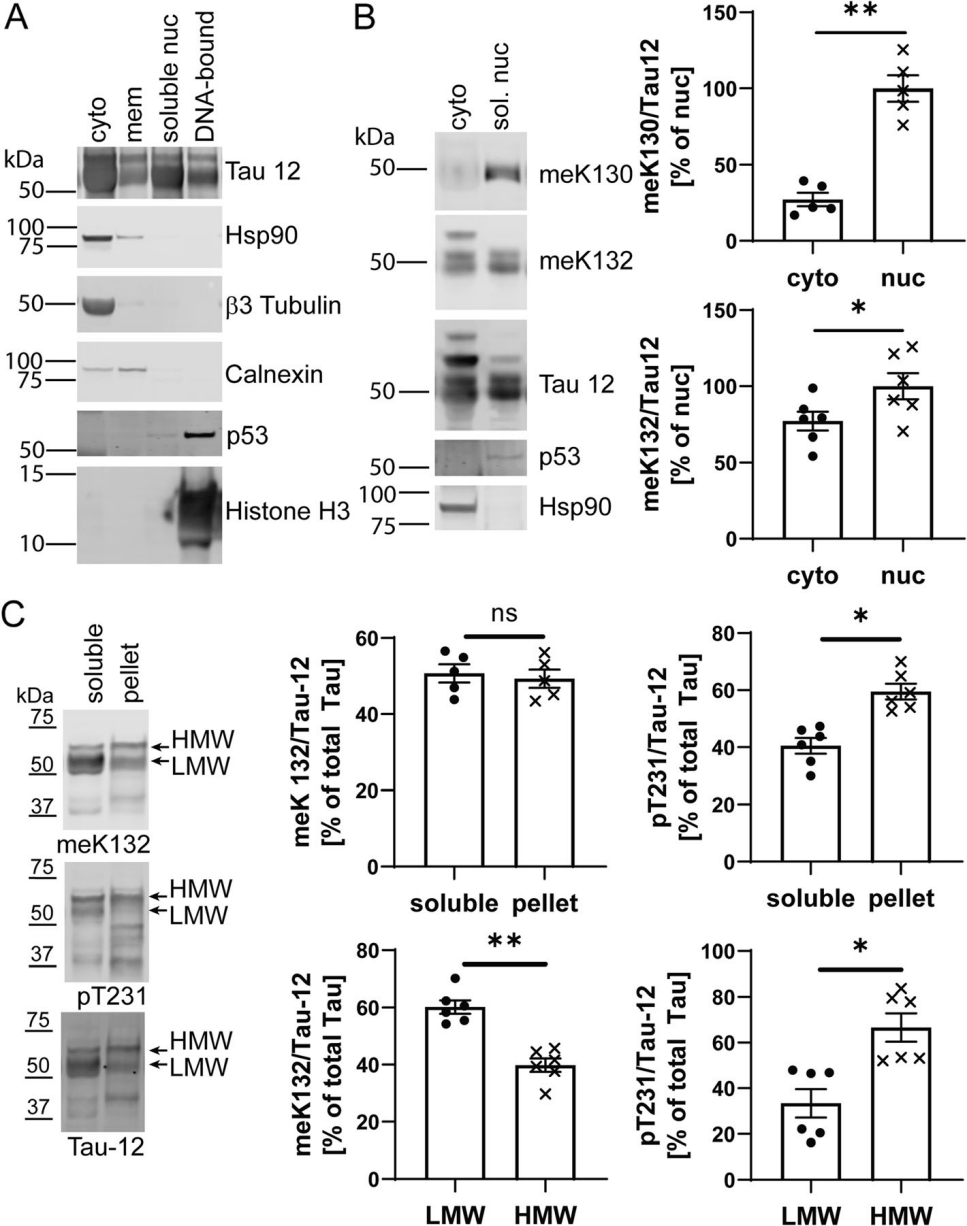
Nuclear, low-molecular weight Tau is preferentially methylated. **A** Cortical tissue from 30 week old rTg4510 mice was subjected to subcellular fractionation and the distribution of Tau was analyzed by Western blotting. The purity of fractions was determined by analyzing the distribution of marker proteins. As demonstrated by a representative blot, the soluble nuclear fraction contains abundant Tau protein but is free from the cytosolic proteins Hsp90, β3-Tubulin and Calnexin. **B** Representative Western blots and quantification demonstrate that meK130 and meK132-modified Tau preferentially localizes to the soluble nuclear fraction in the cortex of 30 week-old rTg4510 mice. **C** Triton-X soluble and insoluble fractions from hippocampi of 30 week-old rTg4510 mice were analyzed by Western blotting. For the distribution between the soluble and the pellet fraction, both HMW and LMW bands were quantified. While pT231-modified Tau is more abundant in the pellet and the HMW fraction, meK132-modified Tau is evenly distributed between soluble fraction and pellet and more abundant on LMW Tau. Statistical significance in (**B**) and (**C**) was determined by paired two-tailed Student’s t-test. *: *p* < 0.05, **: *p* < 0.01

Monomethylation is a small, charge-neutral PTM, and in addition to its role in histone modification it is also a common mechanism to regulate the nuclear translocation of transcription modulators [38]. We therefore investigated the subcellular localization of meK130- and meK132-modified Tau in cortical brain extracts from rTg4510 mice. Biochemical fractionation studies revealed that most of the Tau protein localizes to the cytoplasmic and the soluble nuclear fractions (Fig. 3a). Interestingly, meK130-modified Tau was almost exclusively present in the soluble nuclear fraction in these samples (Fig. 3b). Tau methylated at K132 was present in both the cytoplasmic and the soluble nuclear fractions, with a small but statistically significant enrichment in the nuclear fraction (Fig. 3c).

Insoluble Tau species that accumulate with age in rTg4510 mice are hyperphosphorylated [21], and we consistently observed Tau bands migrating at a larger molecular weight in brain samples from 30 week old mice (Suppl. Fig. S4). These high molecular weight (HMW) species can be removed by treatment with phosphatase, which also abolishes the binding of a phospho-specific antibody to low molecular weight (LMW) Tau (Suppl. Fig. S4). Methylation was not affected by the phosphatase treatment (Suppl. Fig. S4).

To better understand the relationship between methylation and phosphorylation, we next compared the abundance of phospho- and methyl- Tau species in the soluble and pellet fractions obtained from Triton-X 100-extracted hippocampal tissues at 30 weeks of age. As expected, the ratio of phospho/total Tau was higher in the pellet fraction compared to the supernatant, as exemplified by immunoblotting for a phospho-epitope present in Tau aggregates (pT231), while meK132-modified Tau was distributed evenly between the soluble fraction and the pellet (Fig. 3c). Furthermore, the HMW band stained more intensely with the pT231 antibody, an effect that was particularly strong in the pellet fraction. meK132 reactivity on the other hand showed an opposite pattern, with significantly less staining in the HMW compared to the LMW species (Fig. 3c), suggesting that methylation and hyperphosphorylation may not occur on the same species. This is in agreement with our findings that meK130 and meK132-modifications are more prevalent on soluble Tau in both human and mouse brain.

### The lysine methyltransferase SETD7 is specifically localized to the neuronal cytoplasm

**Fig. 4:**
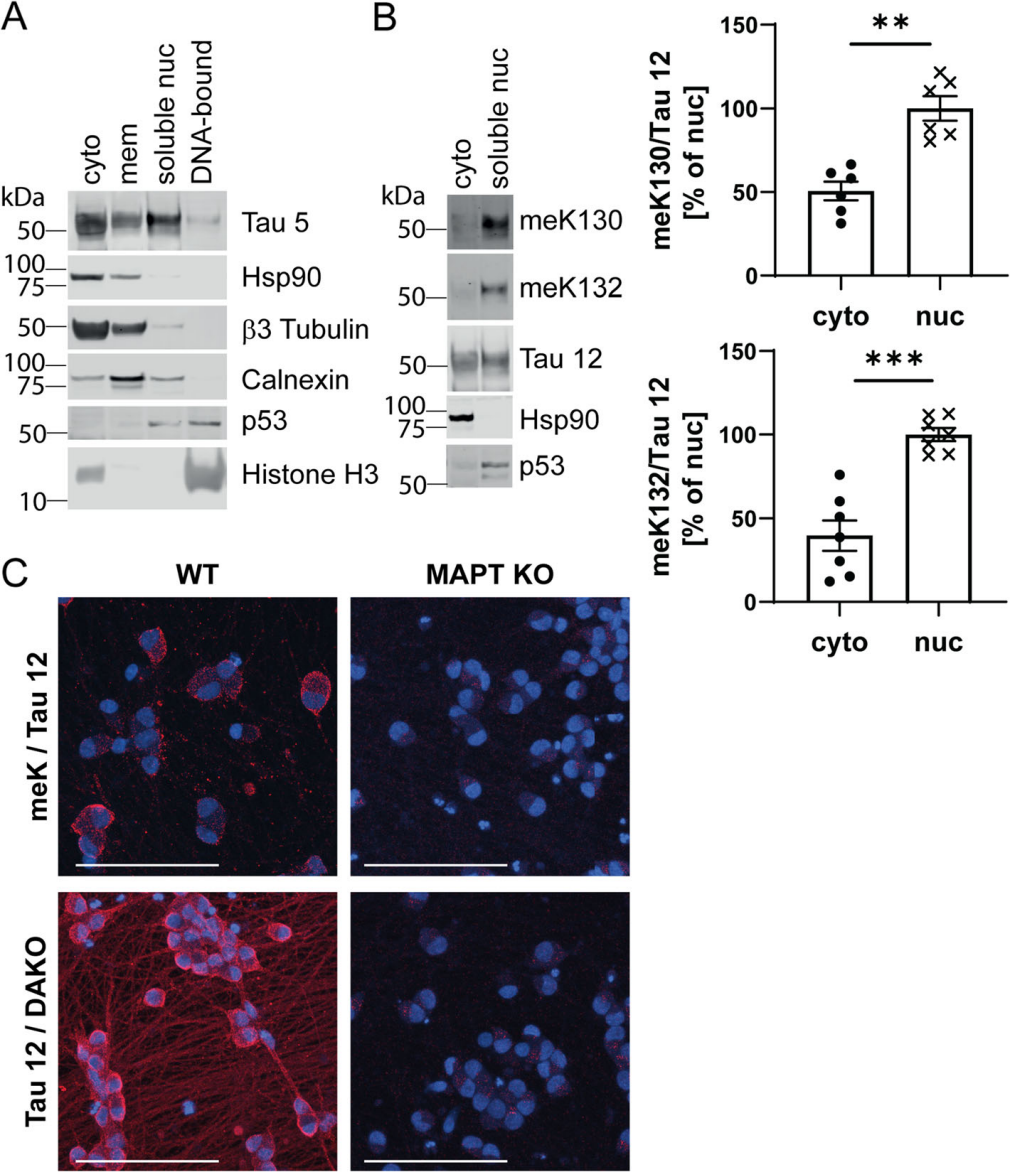
Methylated Tau preferentially localizes to the nucleus. **A** hiPSC-derived neurons were subjected to subcellular fractionation and the distribution of Tau was analyzed by Western blotting. The purity of fractions was determined by analyzing the distribution of marker proteins. Similar to in vivo data, the soluble nuclear fraction contains abundant Tau protein but is free from the cytosolic protein Hsp90. **B** Representative Western blots and quantification demonstrate that meK130 and meK132-modified Tau preferentially localize to the soluble nuclear fraction in hiPSC-derived neurons. **C** hiPSC-derived neurons were subjected to proximity-ligation assays (PLA) using either a total methyl-lysine antibody (meK) in combination with Tau 12, or two total Tau antibodies (Tau 12 and DAKO). Samples were imaged on a confocal microscope. MAPT WT neurons show somatic and nuclear localization of methylated Tau, with additional neuritic staining only observed with the total Tau antibody pair. No signal was observed in MAPT KO neurons. Scale bars: 100 μm. Statistical significance in (**B**) was determined by paired two-tailed Student’s t-test. **: *p* < 0.01, ***: *p* < 0.001

We next analyzed the subcellular distribution of Tau in hiPSC-derived neurons. Similar to mouse brain, Tau was enriched in both cytoplasmic and the soluble nuclear fractions (Fig. 4a), with both meK130 and meK132-modified Tau predominantly found in the soluble nuclear fraction in these samples (Fig. 4b). While our antibodies were not suited for immunofluorescence applications, we took advantage of an antibody recognizing monomethylated lysines (meK) and combined it with a total Tau antibody in a proximity ligation assay (PLA) in hiPSC-derived neurons. The PLA method relies on the spatial proximity of two antibodies, and can be used to detect PTMs, protein-protein interactions as well as protein oligomerization [39]. With the antibody combination meK and Tau 12, we observed a predominantly somatic and nuclear distribution of the PLA signal (Fig. 4c), while the control reaction using two total Tau antibodies (Tau 12 and DAKO against the N- and C-terminus, respectively) showed the expected somatic and neuritic signal (Fig. 4c). Background staining in neurons with a MAPT KO genotype was negligible, confirming the specificity of the assay.

The enzymes involved in protein lysine methylation and demethylation have mainly been described in the nucleus. Their major known substrates are histones, even though additional, non-histone substrates continue to be discovered [38, 40]. For Tau, however, the protein lysine methyl transferase (PKMT) is unknown [41]. We next investigated the expression pattern and subcellular localization of PKMTs in neurons. To this end, we subjected the cytoplasmic, soluble nuclear and DNA-bound fractions from hiPSC-derived neurons to a mass spectrometry-based proteomics analysis and searched for PKMTs among the proteins identified in each fraction. As expected, the majority of PKMTs (15 out of 20) were in the soluble nuclear or DNA-bound fractions, and 4 PKMTs were found in both cytoplasmic and soluble nuclear fractions (Suppl. Fig. S5 and Suppl. Table S4). Only one PKMT (SETD7, SET Domain Containing 7) showed exclusive cytoplasmic localization (Suppl. Fig. S5 and Suppl. Table S4). The Tau sequence at aa130–132 matches the consensus motif for SETD7 (R/KSK(me), [34]), and we therefore decided to investigate this methyl transferase in more detail.

Consistent with the findings from hiPSC-derived neurons, SETD7 localized to the cytoplasmic fraction of both WT and rTg4510 mouse brains (Suppl. Fig. S6), with similar abundance in the two mouse strains (Suppl. Fig. S6). Differential gene expression in human brain was analyzed in two studies from the AMP-AD consortium. The analysis of a public RNAseq data set generated from the Mount Sinai Brain Bank (MSBB) cohort [31] suggested that SETD7 expression is slightly increased in the parahippocampal cortex of demented patients (clinical dementia rating (CDR) 3–5) compared to both patients with mild cognitive impairment (MCI, CDR 1–2) and non-demented donors (CDR 0–0.5), but fold changes (FC) are small (FC 1.15, false discovery rate (FDR) 0.005 for demented vs. MCI, FC 1.13 and FDR 0.012 for demented vs. non-demented, Suppl. Fig. S7A). In this cohort, no difference in SETD7 expression was observed in any of the other brain regions analyzed (Interior Frontal Gyrus (IFG), Fronto Polar Prefrontal Cortex (FP), Superior Temporal Gyrus (STG)) or between Braak stages or between AD and control samples.

Analysis of SETD7 expression in a second large cohort of AD patients from the Mayo clinic [30] showed a slightly increased expression in patients with higher Braak stages (Braak V-VI vs. 0-II: FC 1.33 and FDR 0.0002, Braak V-VI vs. III-IV: FC 1.2 and FDR 0.032), as well as a difference between AD and control samples (FC 1.23 and FDR 0.015, Suppl. Fig. S7B + C). No significant differences were observed in the cerebellum. Taken together, these findings indicate a rather small increase of SETD7 expression in AD, which is partially consistent between two large study cohorts. Potential effects of SETD7 on Tau methylation are thus likely not due to its increased expression in the disease state.

### SETD7 is a methyl transferase for tau

**Fig. 5:**
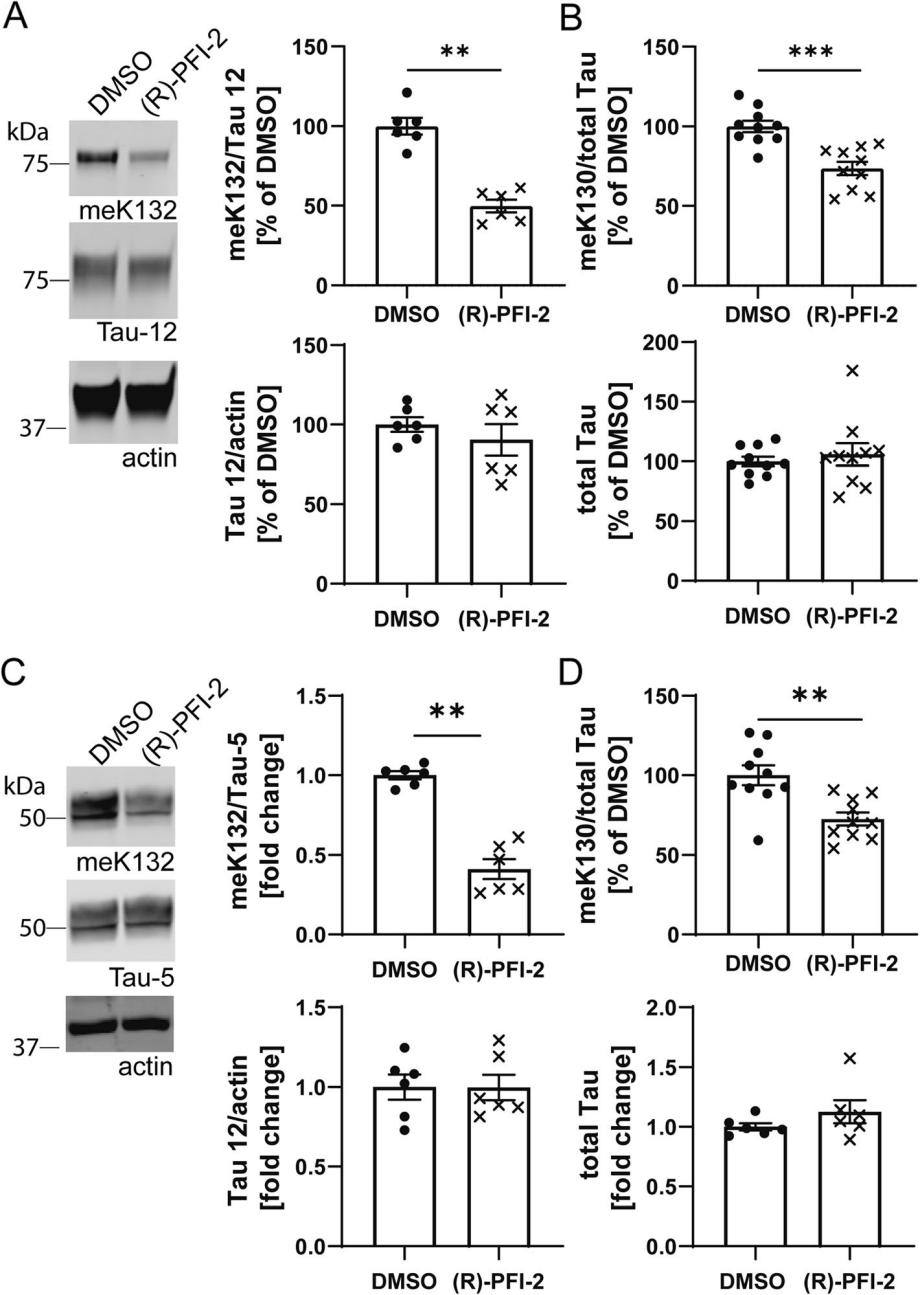
The SETD7 inhibitor (R)-PFI-2 reduces Tau methylation at K130 and K132. **A** Representative Western blots and quantification demonstrate a significant decrease in meK132-modified Tau after treatment of SHSY5Y cells with 5 μM (R)-PFI-2 for 24 h. Total Tau levels remain unaltered. **B** Electrochemiluminescence ELISA shows that treatment with 5 μM (R)-PFI-2 for 24 h reduces the levels of meK130-modified Tau in SH-SY5Y cells. Total Tau levels remain unaltered. **C** Western blots and quantification demonstrate a significant decrease in meK132-modified Tau after treatment of hiPSC-derived neurons with 5 μM (R)-PFI-2 for 48 h. Total Tau levels remain unaltered. **D** Electrochemiluminescence ELISA shows that treatment with 5 μM (R)-PFI-2 for 48 h reduces the levels of meK130 Tau in iPS-derived neurons. Total Tau levels remain unaltered. Statistical significance for all panels was determined by Mann Whitney test. **: *p* < 0.01, ***: *p* < 0.001

We next took advantage of a selective inhibitor of SETD7, (R)-PFI-2 [28], to determine whether SETD7 plays a role in Tau methylation. Indeed, the treatment of SH-SY5Y neuroblastoma cells stably expressing GFP-0N4R Tau with the selective SETD7 inhibitor (R)-PFI-2 [29] significantly reduced both meK132- and meK130-modified Tau (Fig. 5a-b). The treatment did not reduce total Tau levels (Fig. 5a and b), and inhibitors against other PKMTs [27] did not influence either of the methylation sites (Suppl. Fig. 8A + B). Importantly, none of the inhibitors reduced cell viability at the concentrations tested (Suppl. Fig. 8C).

We further observed a significant reduction of endogenous meK132- and meK130-modified Tau in hiPSC-derived neurons after treatment with (R)-PFI-2 (Fig. 5c-d). Again, total Tau levels remained unaffected by the treatment (Fig. 5c-d). Furthermore, we did not observe any changes in Tau phosphorylation at the disease-relevant epitopes AT8 and AT100 in neurons treated with the SETD7 inhibitor, suggesting that the acute modulation of methylation does not influence phosphorylation (Suppl. Fig. S9). Treatment with (R)-PFI-2 did not change the nuclear levels of overall monomethylated Tau, determined by quantification of the PLA signal obtained with the meK/Tau 12 antibody combination (Suppl. Fig. S10A), and no reduction in nuclear total Tau was observed by Western blot (Suppl. Fig. S10B). It is thus likely that the inhibition of SETD7 only affects a small subset of all methylated sites on Tau, which may not be sufficient to alter its subcellular distribution.

**Fig. 6:**
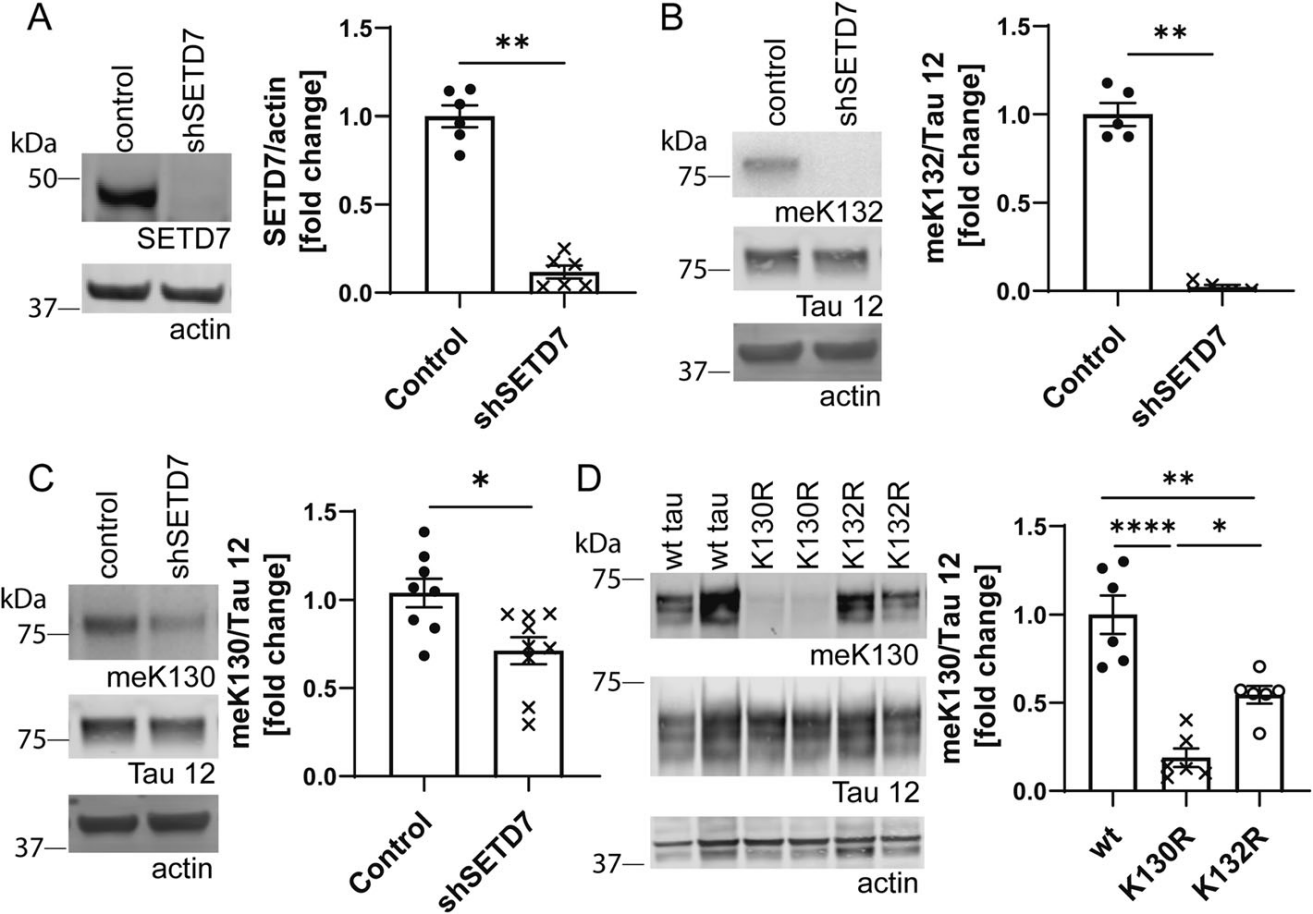
shRNA-mediated knock down of SETD7 expression reduces meK130 and mek132-modified Tau in SHSY5Y cells. **A** Representative Western blots and quantification demonstrate a strong reduction of SETD7 protein in SHSY5Y cells treated with shRNA. **B** meK132-modified Tau is absent in SH-SY5Y cells treated with shRNA against SETD7, representative Western blots and quantification. **C** Representative Western blots and quantification demonstrate that shRNA against SETD7 also reduces the levels of meK130-modified Tau in SH-SY5Y cells, albeit to a smaller degree than the meK132 modification. **D** Transient transfection of WT, K130R and K132R mutant Tau 2N4R into HEK cells demonstrates that the K132R mutation significantly decreases meK130-modified Tau levels. Representative Western blots and quantification are shown. Statistical significance in (**A**), (**B**) and (**C**) was determined by Mann Whitney test, **: *p* < 0.01. Statistical significance in (**D**) was determined by one-way ANOVA (ANOVA *p* < 0.0001), Tukey’s multiple comparisons test was used for post-hoc analysis. *: *p* < 0.05, **: *p* < 0.01, ****: *p* < 0.0001

To exclude potential off-target effects of the pharmacological inhibitor, we next performed a genetic knockdown of SETD7 in SH-SY5Y cells. Using a mix of two lentiviral shRNA constructs, we obtained a reduction of SETD7 protein of app. 90% (Fig. 6a). This was accompanied by reduced levels of both meK132- and meK130-modified Tau in these cells (Fig. 6b and c), confirming the importance of SETD7 for Tau methylation at these residues. Unlike treatment with the pharmacological inhibitor, shRNA mediated knock down of SETD7 also led to a reduction in total Tau levels, possibly due to the longer time-course of the shRNA experiment and the strong reduction of SETD7 protein (Suppl. Fig. S11A).

For both the (R)-PFI-2 and the shRNA knock down, the reduction in methylation was always more pronounced for K132 than K130. The K130 site does not exactly match the consensus sequence for SETD7-mediated methylation (VSK(me) instead of R/KSK(me), [42]). It is therefore possible that K130 is not directly targeted by SETD7, or that methylation at this site requires the initial modification of K132 by SETD7. To investigate the mutual interaction of these sites, we assessed meK130 in the presence of the K132R mutation, which abolishes methylation at aa132 (Fig. 6d and suppl. Fig. S11B). In agreement with our hypothesis we found that the levels of the meK130 modification were significantly reduced for K132R mutant Tau, suggesting that methylation at these sites may be hierarchically regulated and that the inhibition of SETD7 may indirectly reduce the levels of meK130.

## Discussion

Here we report the presence of multiple novel sites for lysine monomethylation on detergent-soluble Tau extracted from human brain samples. Soluble Tau species have been proposed to be responsible for the spreading of Tau pathology throughout the brain during the progression of AD [43], but biochemical signatures differentiating physiological from potentially pathogenic soluble Tau species remain unclear. While Tau methylation is present in both control and AD brain samples, we find that meK130 and meK132 modifications increase with Braak stage. This increase is predominantly observed on soluble Tau protein. In contrast to disease-associated hyperphosphorylation, methylation is less abundant on high-molecular weight Tau, and the inhibition of a Tau PKMT does not influence phosphorylation in vitro. It was shown previously that non-site-specific chemically induced lysine methylation of recombinant Tau prevents fibrillization, both at the nucleation and the elongation steps [12]. Methylation may therefore be a disease-associated modification of Tau that does not promote the formation of NFTs, unlike phosphorylation, proteolytic truncation and acetylation [10, 11, 13, 14, 44, 45], but may co-exist with hyperphosphorylated, aggregation-prone Tau proteoforms.

Most lysine methylation sites that have been identified on Tau so far are situated in the microtubule binding (MTB) domain [12, 36]. In addition to an inhibitory effect on aggregation, methylation may thus lower the affinity of Tau for microtubules and facilitate its retention in selected subcellular compartments. Interestingly, a recent study reports preferential nuclear localization of an N279K Tau mutant associated with frontotemporal dementia, for which the lysine introduced by the mutation falls within the MTB region [46].

Promoting nuclear localization of target proteins is a known function of lysine methylation and has been reported for non-histone targets such as p53 or the estrogen receptor [47, 48]. Here we find that SETD7, which we identify as an important protein lysine methyltransferase (PKMT) for Tau at K130 and K132, resides in the neuronal cytoplasm, in contrast to most other neuronal PKMTs. Indeed, different from other SET domain PKMTs, SETD7 does not carry a nuclear localization signal and its targets can be found in both the cytoplasm and the nucleus [49]. While both meK130- and meK132-modified Tau are predominantly nuclear, the cytoplasmic fraction of rTg4510 mouse brain contained a larger proportion of meK132- than meK130-modified species. While our data show that the inhibition of SETD7 is not sufficient to shift the subcellular localization of Tau, this intervention likely does not affect all methylation sites on Tau. Since our PLA data demonstrate that all monomethylated Tau species reside in the neuronal soma and nucleus and are absent from neurites, it is possible that additional methylation sites play an important role in determining the subcellular localization of Tau.

The Tau sequence preceding the methylation site at K132 corresponds to the consensus sequence for SETD7 [42]. Using an inhibitor as well as an shRNA-mediated knock down approach we confirmed that this enzyme is the main PKMT responsible for generating meK132-modified Tau. Interestingly, inhibition or knock down of SETD7 also reduced Tau methylation at K130, even though this site does not match the SETD7 consensus sequence (VSK(me) instead of R/KSK(me)). The reduction of meK130-modified Tau levels was also consistently smaller compared to the effect of SETD7 inhibition on K132, suggesting that K130 may not be a direct target. We also show that the K132R mutation, which abolishes methylation at K132, reduces the meK130 signal, supporting the hypothesis that the methylation events at these two sites are linked. This observation, together with our mass spectrometry data and other previously published lysine methylation sites in Tau, suggests that the Tau methylome forms a complex regulatory system similar to the regulation of p53 through differential, potentially hierarchical methylation at adjacent sites by multiple PKMTs [50] and may be as complex as Tau phosphorylation.

## Conclusions

This study shows that lysine methylation, similar to phosphorylation, is an abundant PTM on Tau and affects many different sites across the Tau sequence. Like phosphorylation, lysine methylation on certain sites increases with Braak stage, but affects a different pool of Tau molecules. While phosphorylation is linked to misfolding and aggregation of Tau, our data suggest that lysine methylation is associated with soluble Tau and may modulate its subcellular localization. Additional studies on Tau lysine PTMs, their interactions and relevance for Tau function and dysfunction in different neurodegenerative diseases are thus warranted.

## Supplementary Information

**Additional file 1: Supplementary Figure S1.** Methyl-Tau antibodies specifically recognize methylated over non-modified Tau peptides. Non-modified recombinant Tau 2N4R, Tau 2N4R subjected to reductive methylation as well as methylated and non-methylated Tau-derived peptides were spotted onto nitrocellulose membranes and probed with the different methyl-Tau antibodies used in this study. **Supplementary Figure S2.** Three of five methyl-Tau antibodies demonstrate specificity for their methylation site in a cell lysate context. HEK293T cells were transiently transfected with wt 2N4R Tau or mutant Tau proteins (K130R, K132R, K343R, K353R or K438R). While the antibodies directed against meK130, meK132 and meK353 specifically recognize wt, but not the corresponding mutant Tau, antibodies against meK343 and meK438 show no such specificity. **Supplementary Figure S3.** Sarkosyl Extraction scheme. **Supplementary Figure S4.** Treatment with λ-phosphatase abolishes the HMW Tau band. Cytosolic and soluble nuclear fractions derived from 8- and 30-week old tg4510 mouse cortex were subjected to dephosphorylation with λ-phosphatase. Staining with Tau 12 demonstrates that the high molecular weight (HMW) Tau band apparent in 30-week old animals is abolished upon phosphatase treatment. Furthermore, the pT231 antibody staining, which strongly stains the HMW bands in untreated samples, is also removed by the treatment. Stainings with the meK130 and meK132 antibodies demonstrate that Tau methylation is not removed by phosphatase treatment. **Supplementary Figure S5.** Subcellular localization of PKMTs identified by MS proteomics in iPS-derived neurons. **Supplementary Figure S6.** SETD7 levels and subcellular localization in rTg4510 mouse brain. Cortical tissue from 16 week old WT and rTg4510 mice was subjected to subcellular fractionation and Western blotting for SETD7 as well as the fraction markers Hsp90 and HDAC2. In agreement with the data from hiPSC-derived neurons, SETD7 also localizes to the cytoplasmic fraction in mouse brain samples. No statistically significant difference in the abundance of SETD7 between WT and rTg4510 mice was detected upon quantification and normalization to Hsp90. Statistical significance was determined by unpaired two-tailed Student’s t-test, n.s.: not significant. **Supplementary Figure S7.** Differential gene expression of SETD7 in human postmortem brain tissue for multiple AD related phenotypes in two independent cohorts. **(A)** SETD7 gene expression in the Mount Sinai Brain Bank study (AMP-AD) for dementia status. SETD7 is significantly higher expressed in cases with dementia (CDR 3-5) compared to no dementia (CD 0-0.5) or MCI (CDR 1-2). **(B)** SETD7 gene expression in the Mayo study (AMP-AD) for Braak stages. SET7 is significantly higher expressed in brain tissue of patients with Braak V-VI compared to patients with low (0-II) or medium Braak stages (III-IV). **(C)** SETD7 gene expression in the Mayo study (AMP-AD) for AD status. SETD7 is significantly higher expressed in brain tissue of AD patients compared to controls. Please refer to the methods section for details of the analysis. **Suppl. Fig. S8.** The influence of PKMT inhibition on neuronal viability and methylated Tau levels. **(A) – (C)** SH-SY5Y cells were treated with different PKMT inhibitors as described in the methods section. No influence on meK130 and meK132 levels or viability was observed. Statistical significance was determined by one-way ANOVA. ANOVA meK130: *p* = 0.1910, ANOVA meK132: *p* = 0.3367, ANOVA viability: *p* = 0.3750. **Suppl. Fig. S9.** SETD7 inhibitor treatment does not alter Tau phosphorylation in hiPSC-derived neurons. 48h of treatment with 10 μM (R)-PFI-2 does not alter AT8 or AT100 signal, but significantly decreases meK132 Tau signal as measured by ELISA. Statistical significance was determined by unpaired two-tailed Student’s t-test, n.s.: not significant, **: *p*<0.01. **Suppl. Fig. S10.** (R)-PFI-2 treatment does not change nuclear Tau levels in hiPSC neurons. hiPSC-derived neurons were treated for 48h with 10 μM (R)-PFI-2, and levels of nuclear, overall monomethylated Tau were determined by PLA with the meK and the Tau 12 antibodies and confocal microscopy **(A)**. The levels of total, nuclear Tau were determined by subcellular fractionation and Western blotting **(B)**. Scale bars: 100 μm. Statistical significance was determined by unpaired two-tailed Student’s t-test, n.s.: not significant. **Suppl. Fig. S11.** The influence of SETD7 knockdown and K/R mutations on total Tau levels. **(A)** shRNA treatment against SETD7 leads to a slight reduction in total Tau levels in SH-SY5Y cells. For a representative Western blot, see Fig. 6C. **(B)** Total Tau levels do not significantly differ in HEK293T cells transiently transfected with wt 2N4R Tau or the K130R and K132R mutant proteins. For a representative Western blot, see Fig. 6D. Statistical significance was determined by Mann-Whitney test in **(A)**, and one-way ANOVA in **(B)**. **: p<0.01, ANOVA *p*=0.8096.

**Additional file 2: Supplementary S12.** Full blots.

**Additional file 3: Suppl Table S1.** Characteristics of human brain samples used for Tau PTM discovery. **Suppl. Table S2**. PKMT inhibitors and concentrations used in cell culture. **Suppl. Table S3.** PTM modifications identified on tau protein immunoprecipitated from human entorhinal cortex. Trypsin and AspN digests of tau were analyzed by LC-MS/MS and resulting m/z data was analyzed by MASCOT using 10 ppm mass tolerance for the search. ^1^amino acid (aa) residue numbering conforms to human 2N4R tau, except for peptide 68-97 (pS113). ^2^aa residue according to human 1N4R tau. ^3^K modified lysine, S modified serine, T modified threonine. ^4^Enzyme used to generate peptides for MS. ^5^modified site according to human 2N4R tau. me: monomethylation, ac: acetylation, p: phosphorylation, MW: molecular weight in Dalton. **Suppl. Table S4.** Characteristics of human brain samples used for sarkosyl extraction. **Suppl. Table S5.** Subcellular localization of PKMTs identified by MS.

**Additional file 4: Suppl. Table S6.** Peptide counts in subcellular fractions

## Data Availability

The mass spectrometry datasets generated and analysed during the current study are available in the ProteomeXchange Consortium via the PRIDE [26] partner repository with the dataset identifier PXD017065.

## Abbreviations

AD: Alzheimer’s disease
CDR: Cognitive dementia rating
DMSO: Dimethyl sulfoxide
ELISA: Enzyme-linked immunosorbent assay
FC: Fold change
FDR: False discovery rate
FP: Fronto polar prefrontal cortex
hiPSC: human induced pluripotent stem cell
HMW: High molecular weight
IFG: Interior frontal gyrus
KO: Knock out
LMW: Low molecular weight
MCI: Mild cognitive impairment
meK: methyl lysine
MS: Mass spectrometry
MSBB: Mount Sinai brain bank
NFT: Neurofibrillary tangle
PKMT: Protein lysine methyltransferase
PLA: Proximity ligation assay
PTM: Post-translational modification
SETD7: SET-domain containing 7
shRNA: short hairpin RNA
STG: Superior Temporal Gyrus
WT: Wild-type
