# Supplementary material for: SETD7-mediated monomethylation is enriched on soluble Tau in Alzheimer’s disease
Source: Mol Neurodegener. 2021 Jul 2;16:46. doi: 10.1186/s13024-021-00468-x (PMC8254302; doi:10.1186/s13024-021-00468-x)
Supplement: Supplementary file 1 — Additional file 1: Supplementary Figure S1. Methyl-Tau antibodies specifically recognize methylated over non-modified Tau peptides. Non-modified recombinant Tau 2N4R, Tau 2N4R subjected to reductive methylation as well as methylated and non-methylated Tau-derived peptides were spotted onto nitrocellulose membranes and probed with the different methyl-Tau antibodies used in this study. Supplementary Figure S2. Three of five methyl-Tau antibodies demonstrate specificity for their methylation site in a cell lysate context. HEK293T cells were transiently transfected with wt 2N4R Tau or mutant Tau proteins (K130R, K132R, K343R, K353R or K438R). While the antibodies directed against meK130, meK132 and meK353 specifically recognize wt, but not the corresponding mutant Tau, antibodies against meK343 and meK438 show no such specificity. Supplementary Figure S3. Sarkosyl Extraction scheme. Supplementary Figure S4. Treatment with λ-phosphatase abolishes the HMW Tau band. Cytosolic and soluble nuclear fractions derived from 8- and 30-week old tg4510 mouse cortex were subjected to dephosphorylation with λ-phosphatase. Staining with Tau 12 demonstrates that the high molecular weight (HMW) Tau band apparent in 30-week old animals is abolished upon phosphatase treatment. Furthermore, the pT231 antibody staining, which strongly stains the HMW bands in untreated samples, is also removed by the treatment. Stainings with the meK130 and meK132 antibodies demonstrate that Tau methylation is not removed by phosphatase treatment. Supplementary Figure S5. Subcellular localization of PKMTs identified by MS proteomics in iPS-derived neurons. Supplementary Figure S6. SETD7 levels and subcellular localization in rTg4510 mouse brain. Cortical tissue from 16 week old WT and rTg4510 mice was subjected to subcellular fractionation and Western blotting for SETD7 as well as the fraction markers Hsp90 and HDAC2. In agreement with the data from hiPSC-derived neurons, SETD7 also localizes to the cytopla [file 13024_2021_468_MOESM1_ESM.pdf]

2N4R	recombinant tau
me2N4R	in vitro methylated recombinant tau
meK130	CQARMVSK(me)SKDGTG-NH2
meK132	CRMVSKSK(me)DGTGSD-NH2
meK343	CVEVKSEK(me)LDFKDR-NH2
meK353	CKDRVQSK(me)IGSLDN-NH2
meK438	CDEVSASLAK(me)QGL-NH2
K130/K132	CQARMVSKSKDGTGSD-NH2
K343	CVEVKSEKLDFKDR-NH2
K353	CKDRVQSKIGDLDN-NH2
K438	CDEVSASLAKQGL-NH2

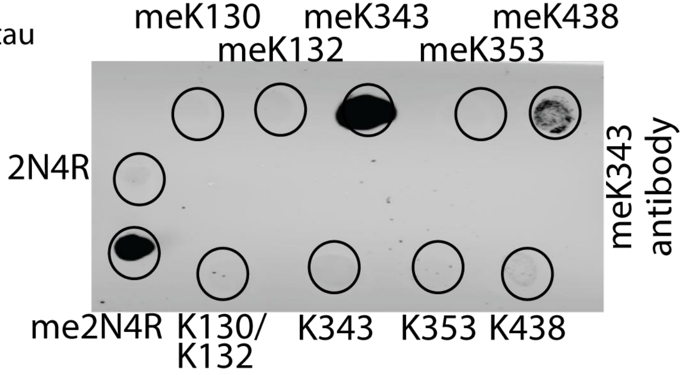

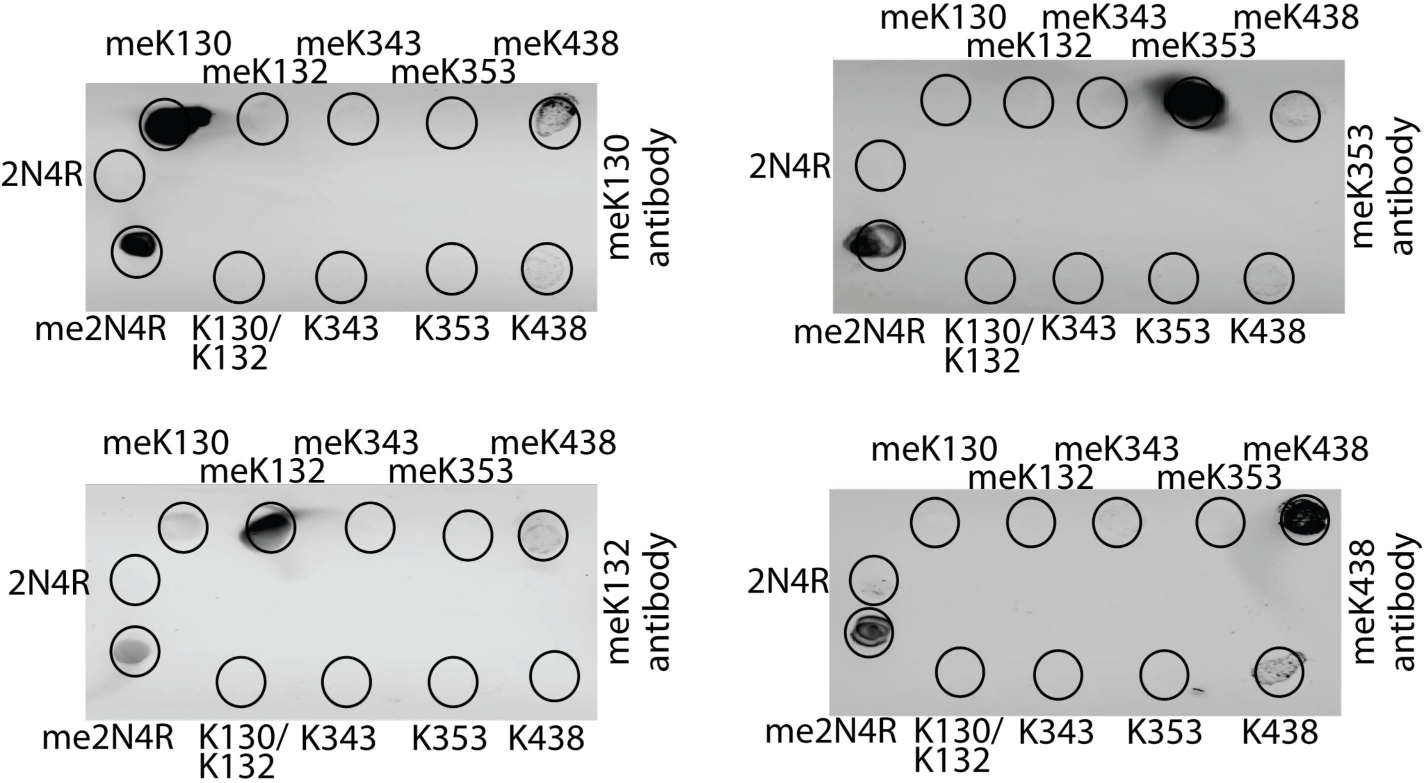

Suppl Figure S1

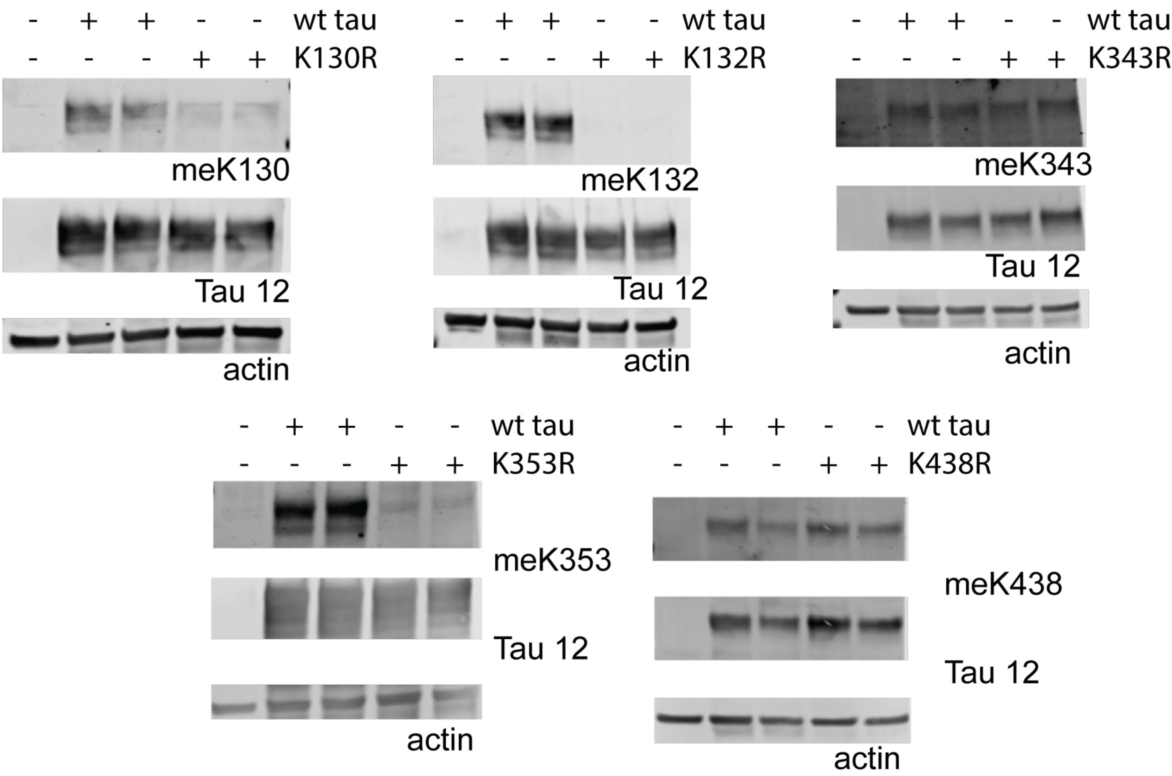

Suppl Figure S2

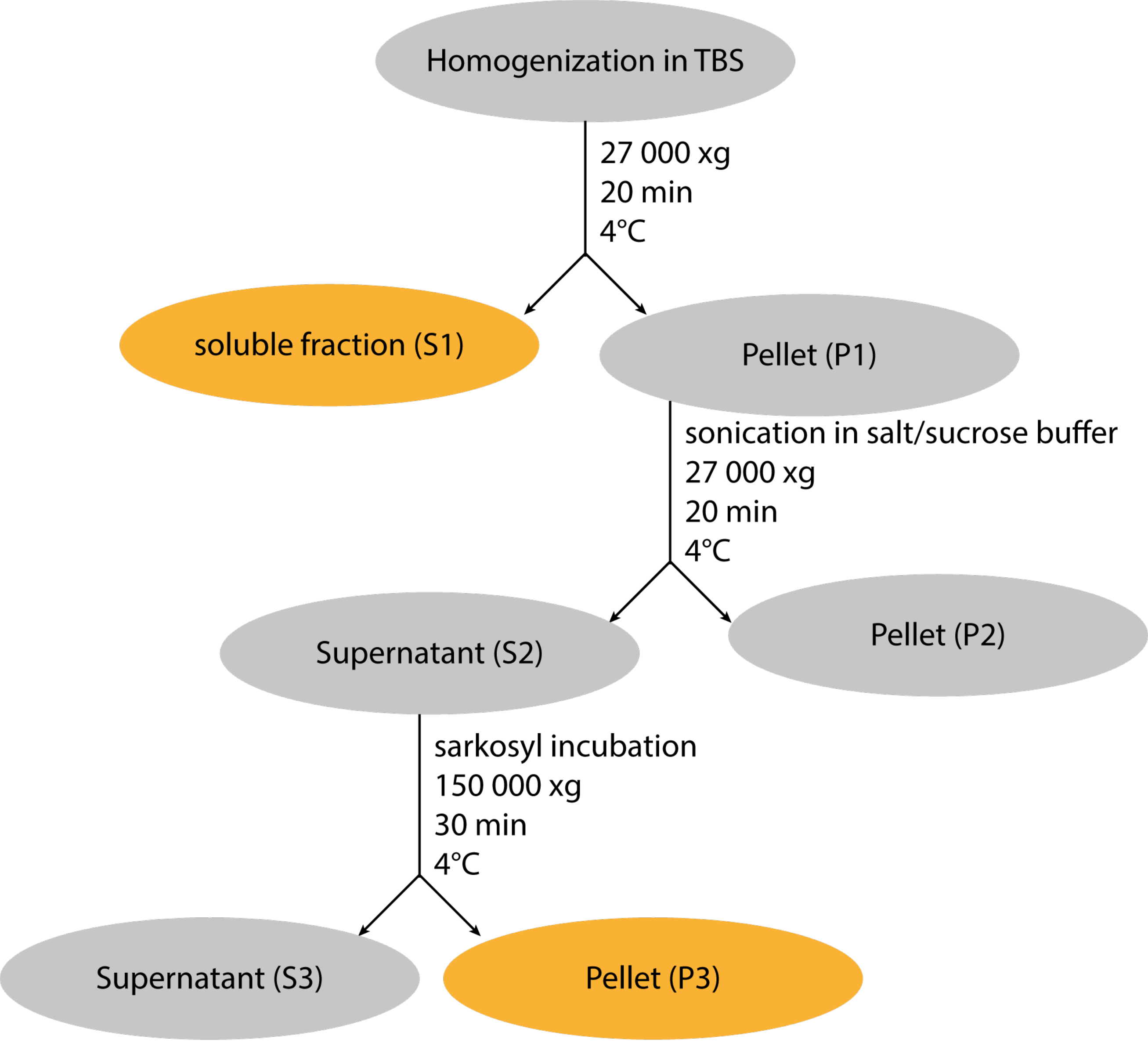

Suppl Figure S3

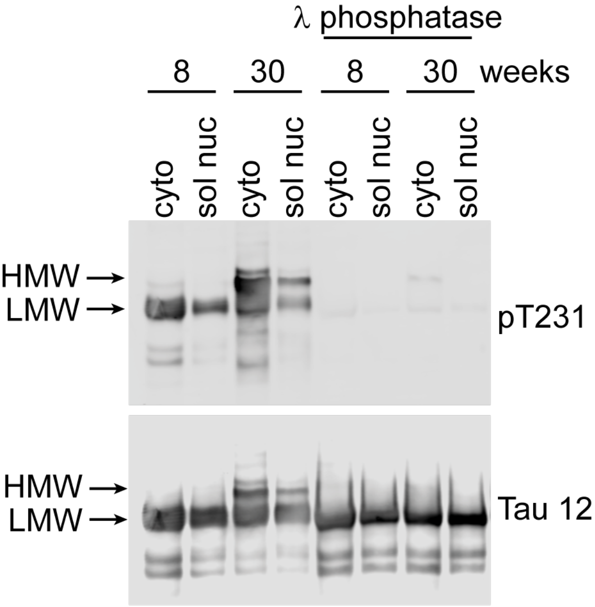

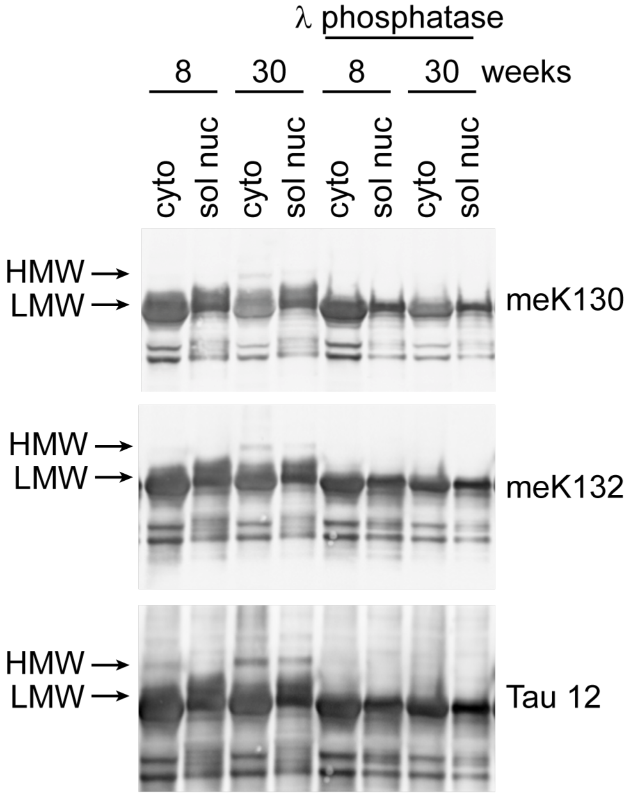

Suppl Figure S4

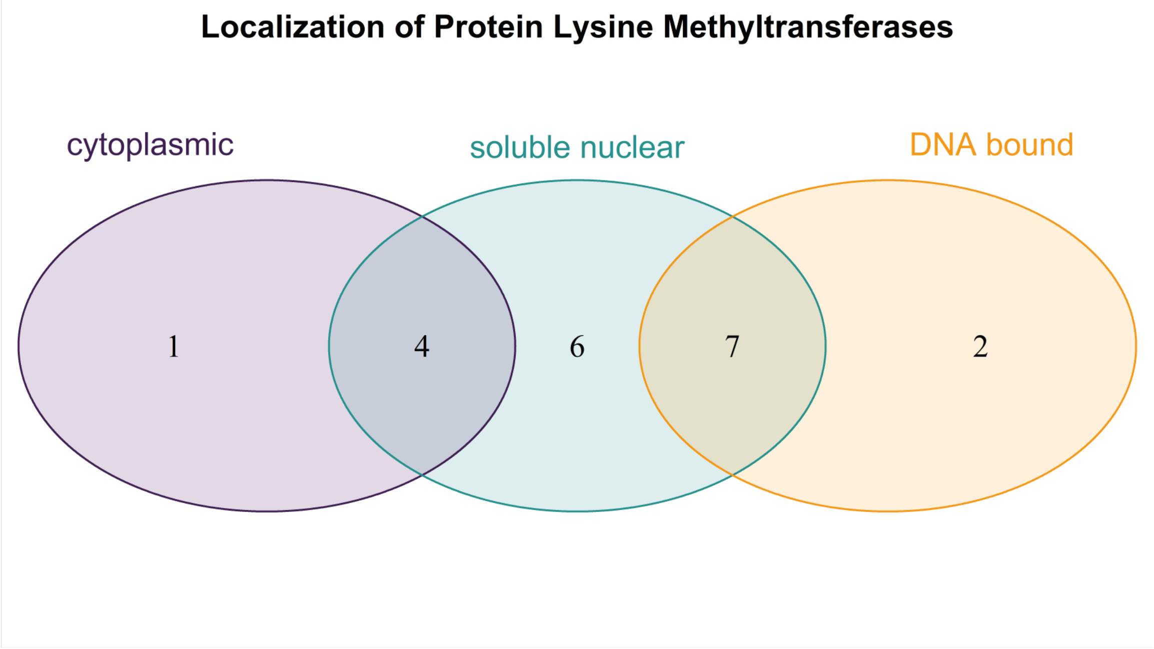

Suppl Figure S5

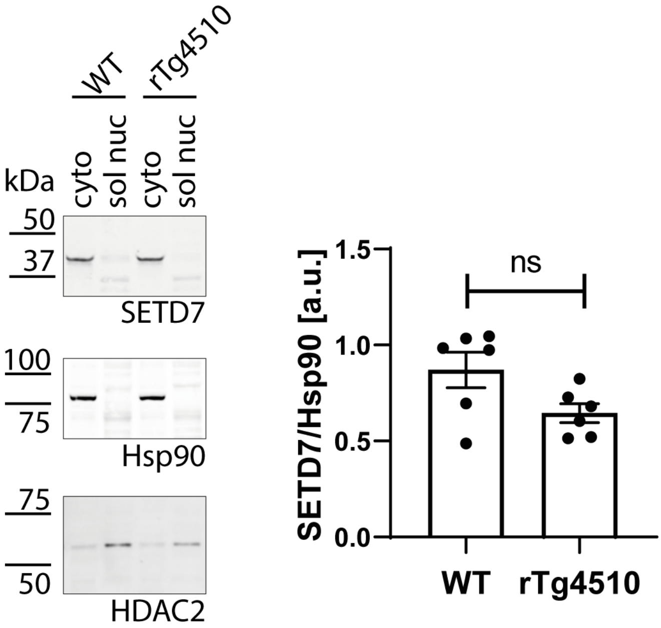

Suppl Figure S6

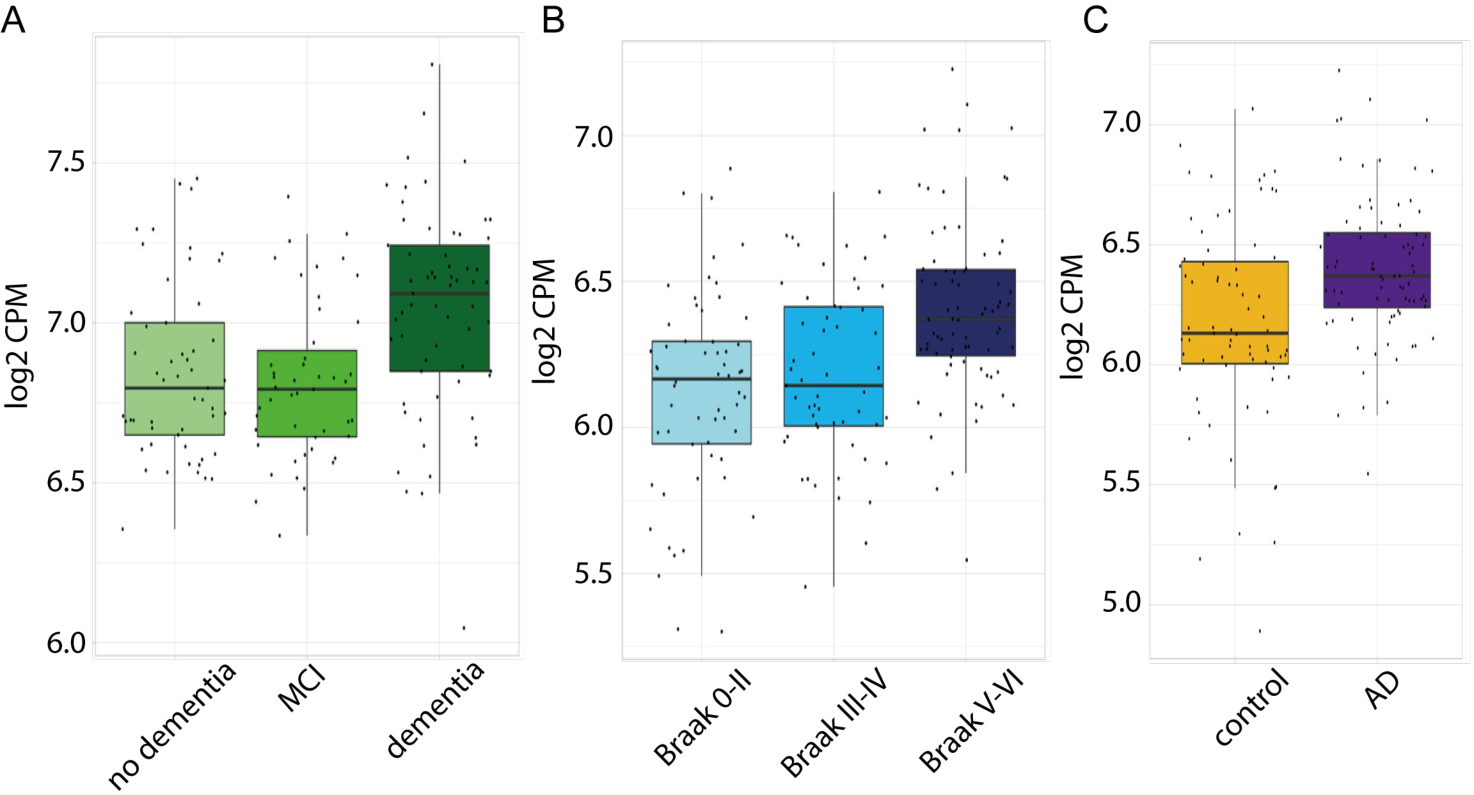

Suppl Figure S7

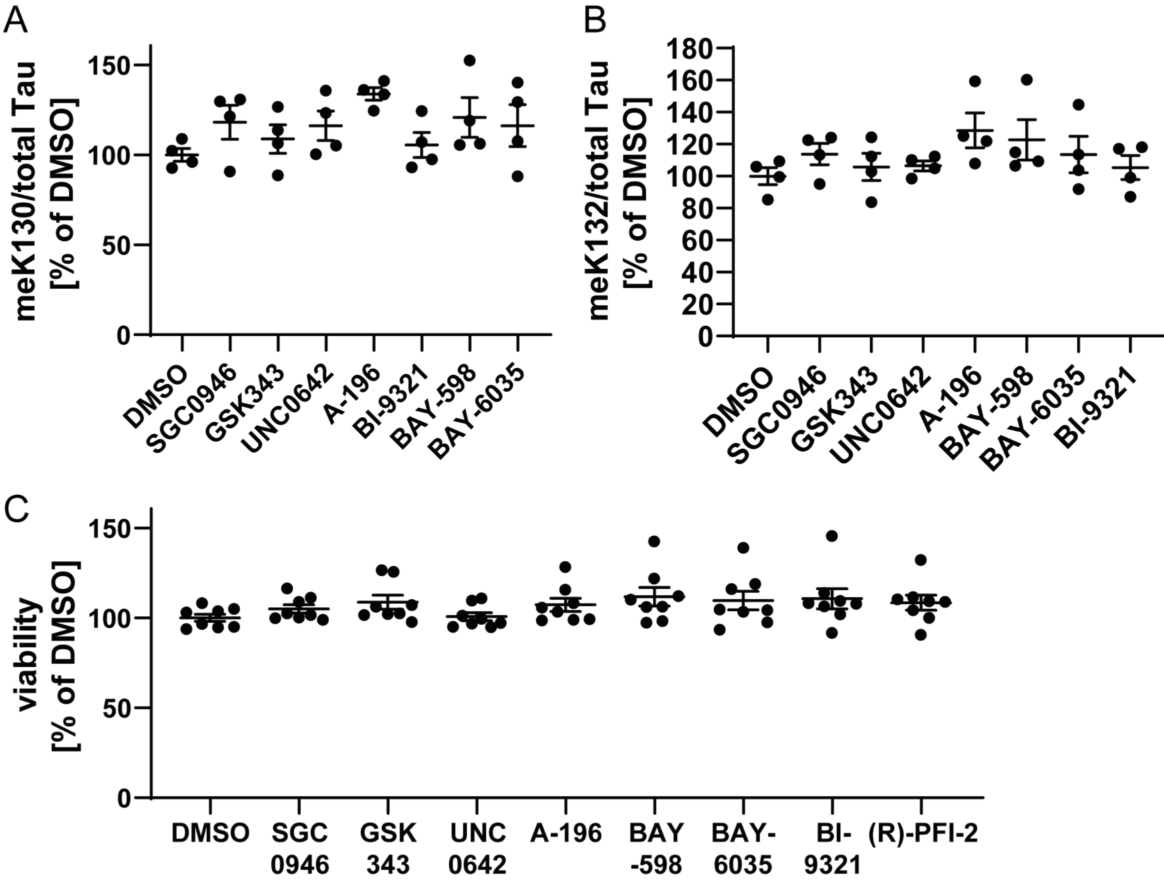

Suppl Figure S8

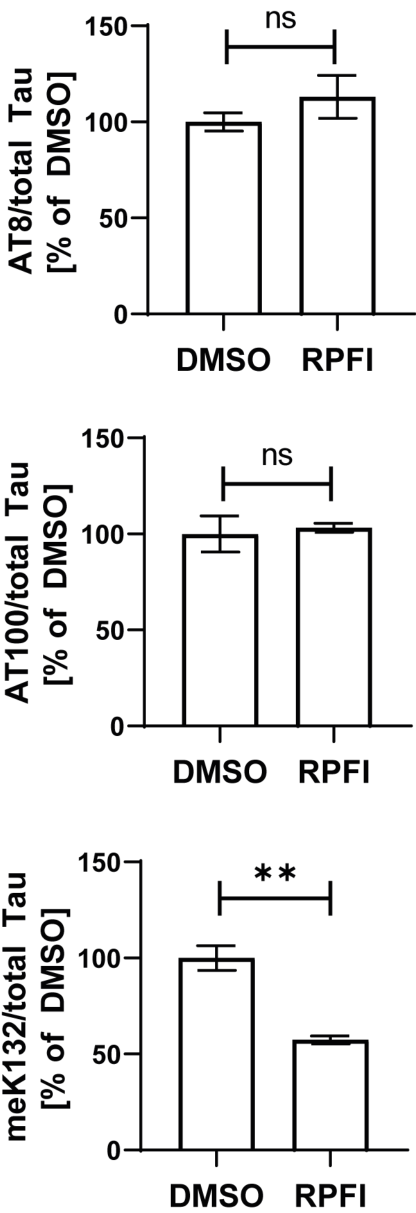

Suppl Figure S9

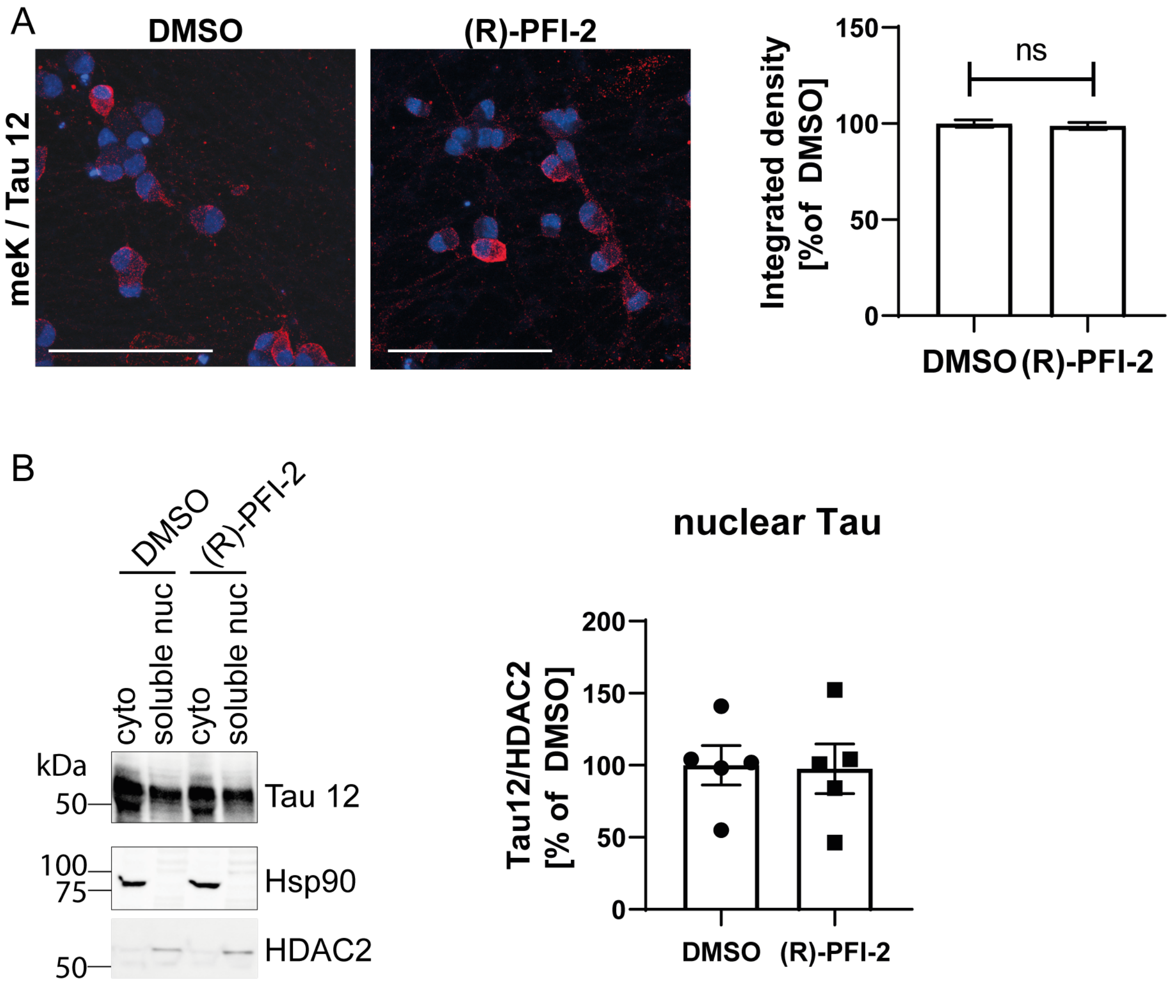

Suppl Figure S10

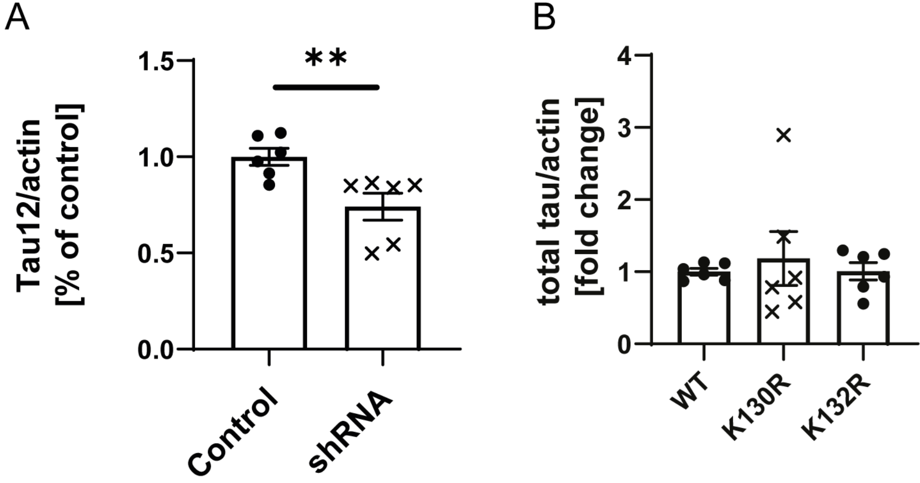

Suppl Figure S11
