# Supplementary material for: SETD7-mediated monomethylation is enriched on soluble Tau in Alzheimer’s disease
Source: Mol Neurodegener. 2021 Jul 2;16:46. doi: 10.1186/s13024-021-00468-x (PMC8254302; doi:10.1186/s13024-021-00468-x)
Supplement: Supplementary file 2 — Additional file 2: Supplementary S12. Full blots. [file 13024_2021_468_MOESM2_ESM.pdf]

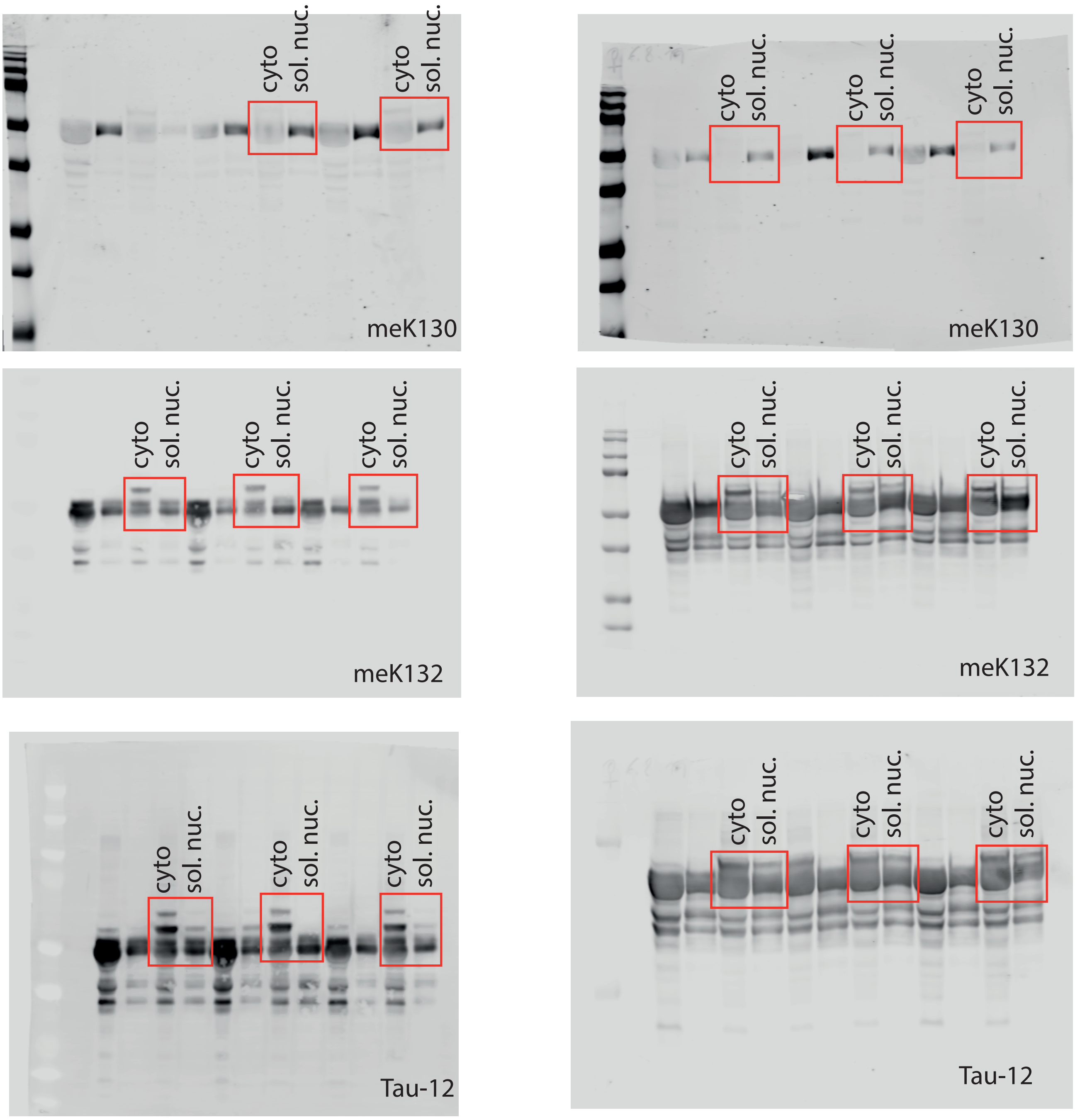

Full Western blot images related to Fig. 3B

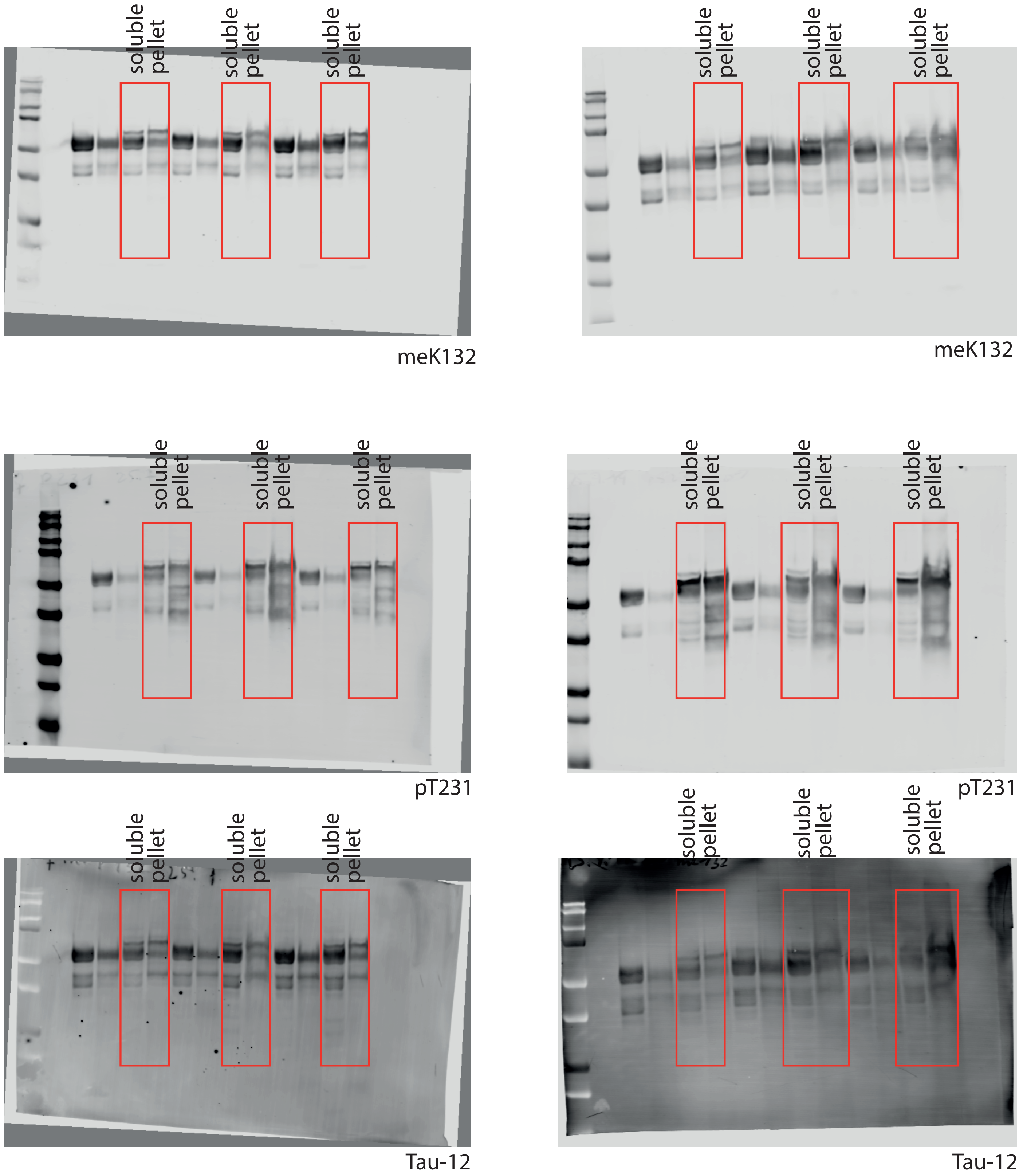

Full Western blot images related to Fig. 3C

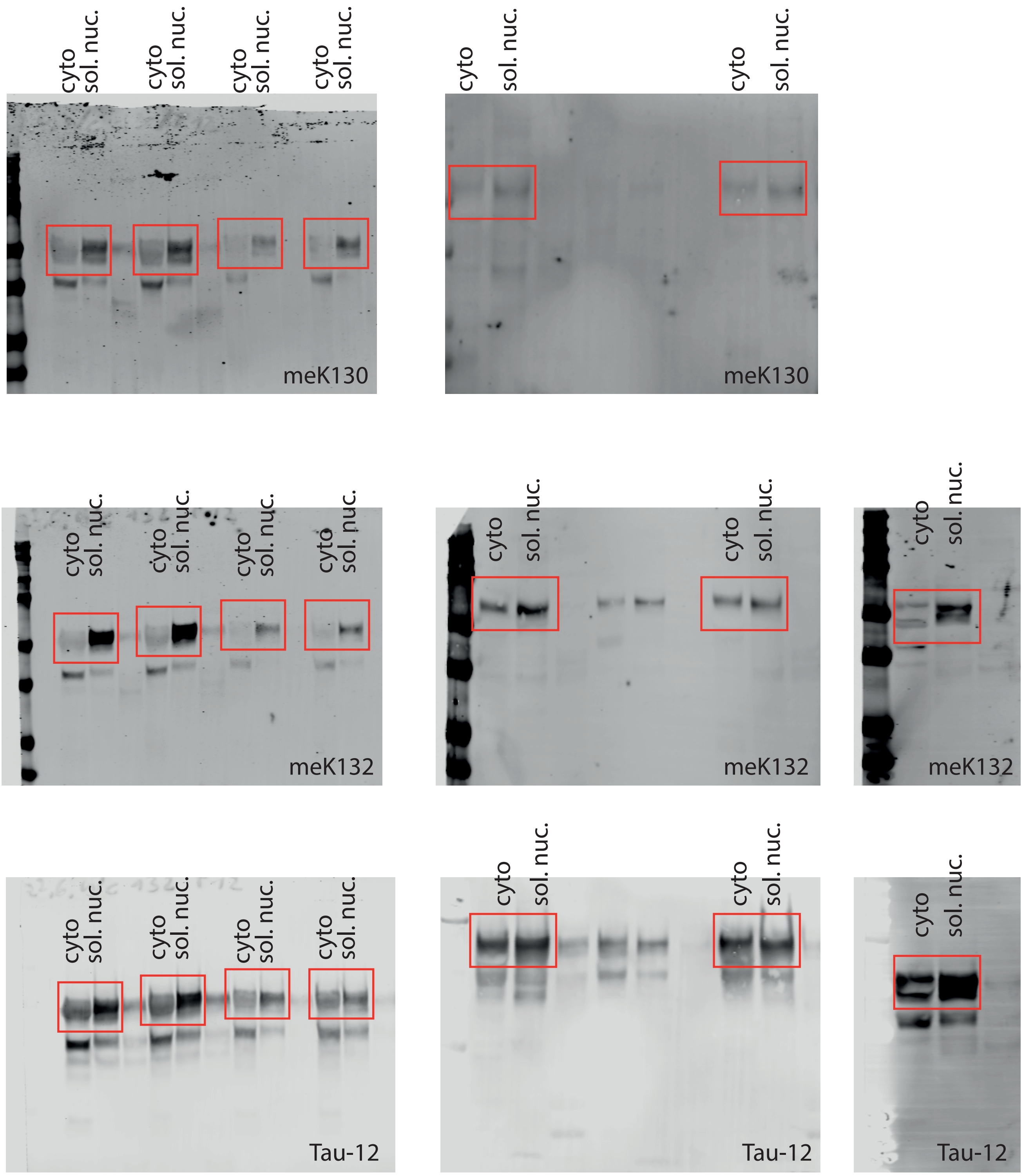

Full Western blot images related to Fig. 4B

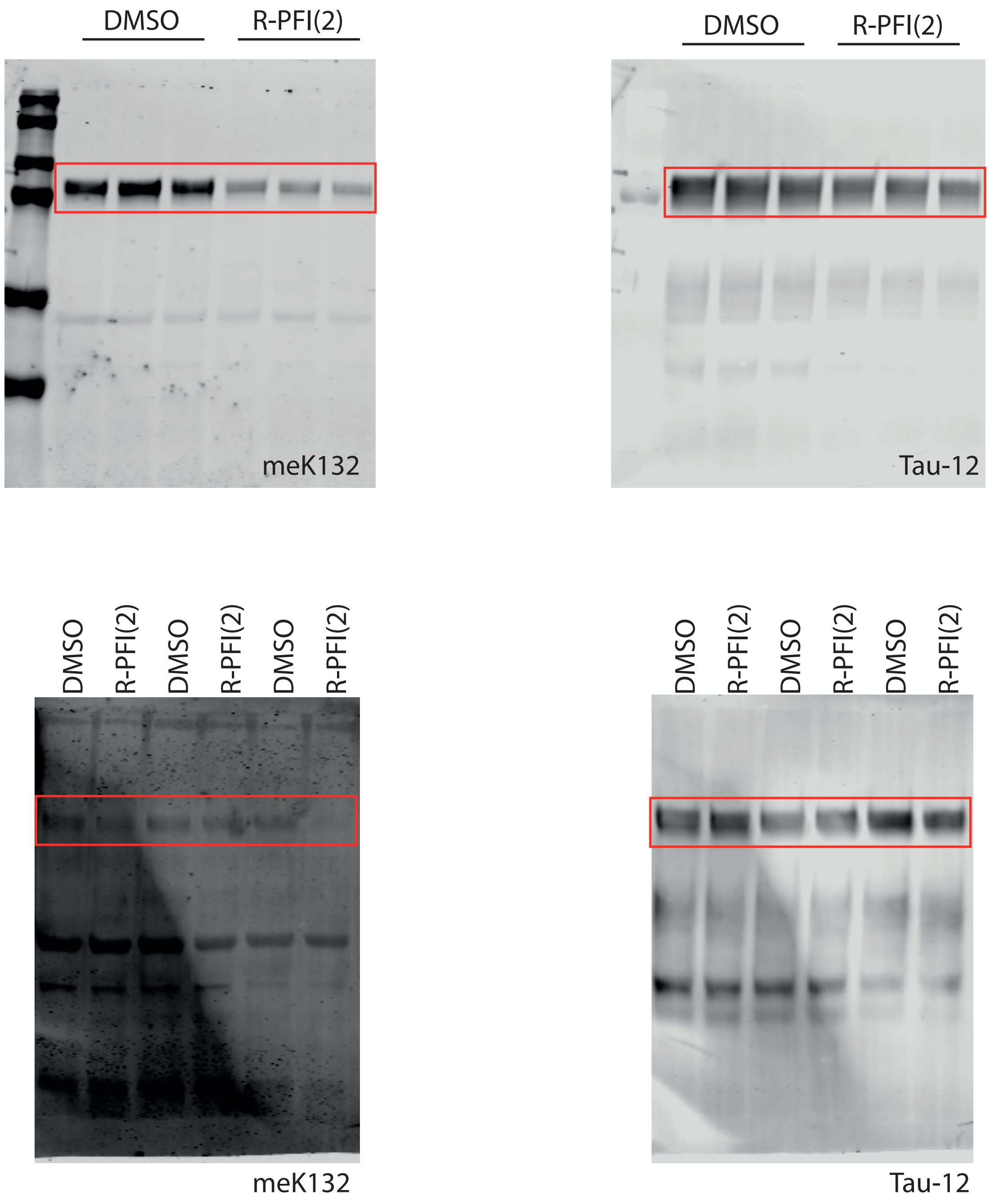

Full Western blot images related to Fig. 5A

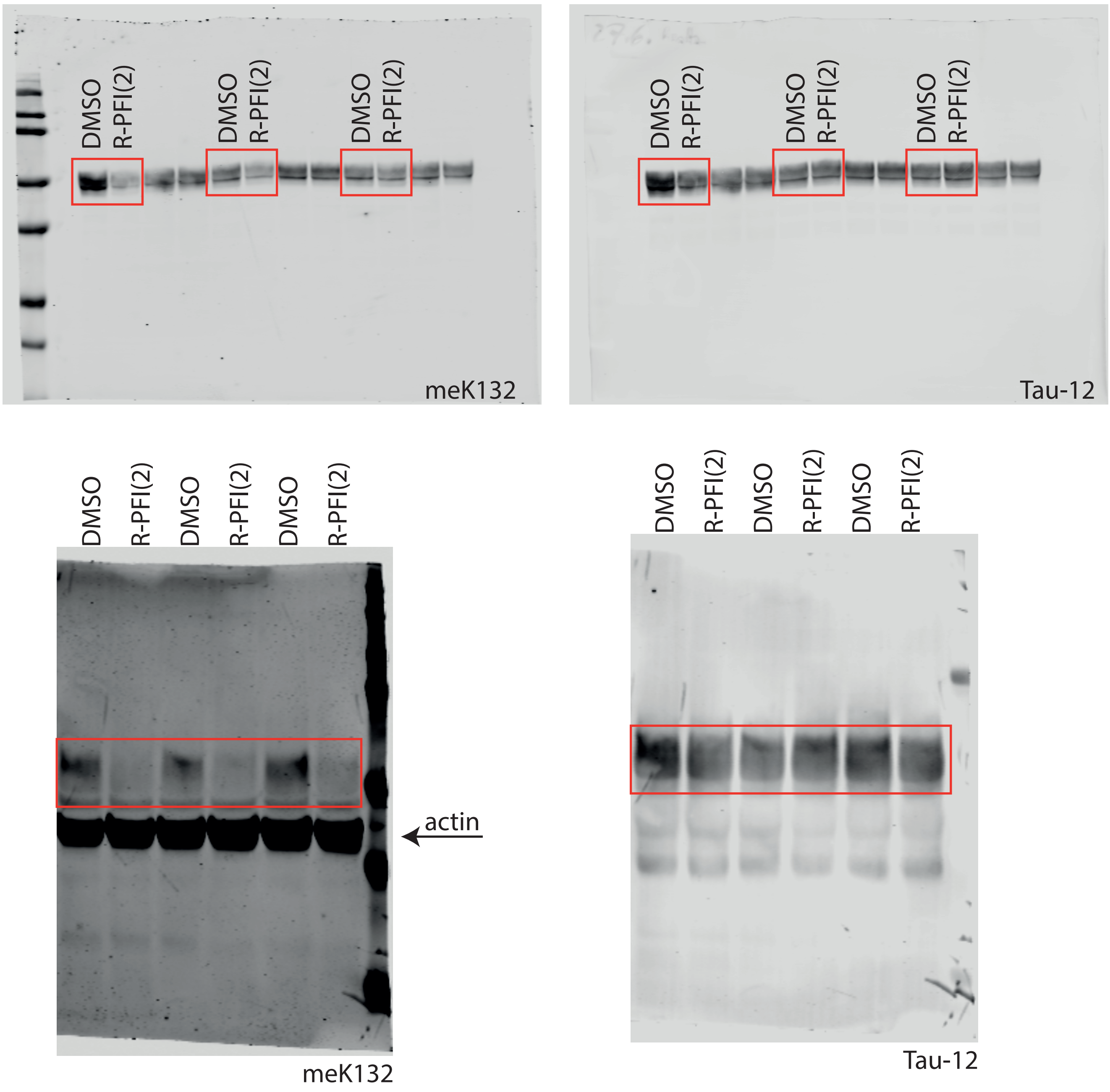

Full Western blot images related to Fig. 5B

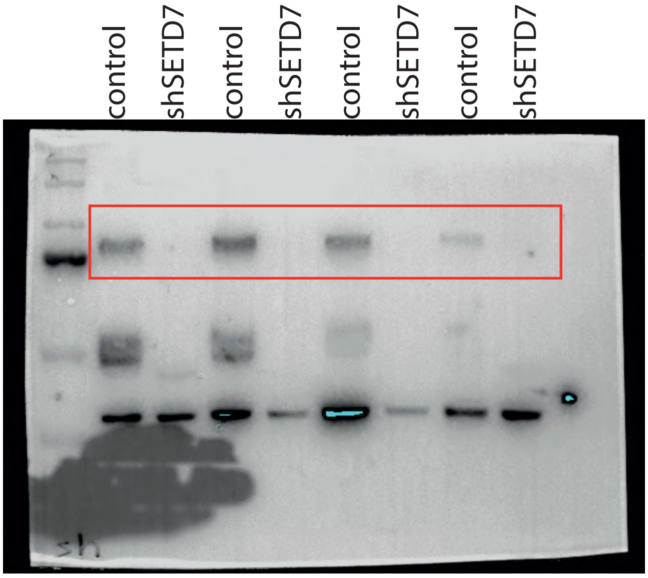

meK132

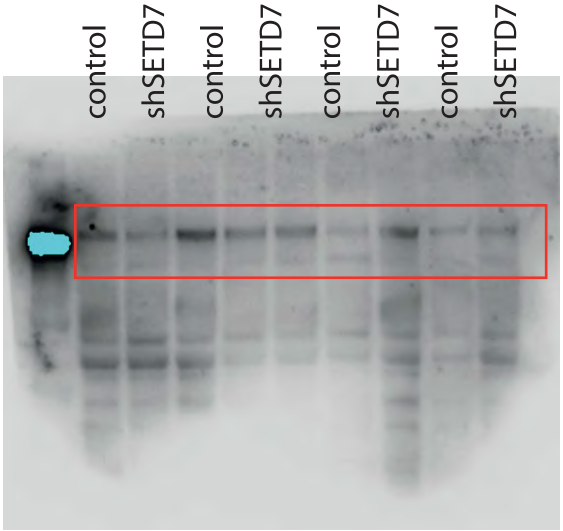

meK130

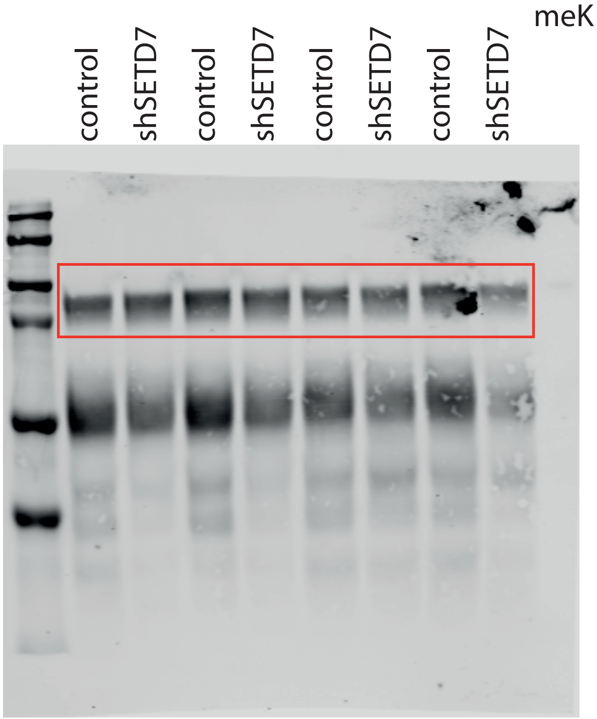

Tau-12

Full Western blot images related to Fig. 6B+C

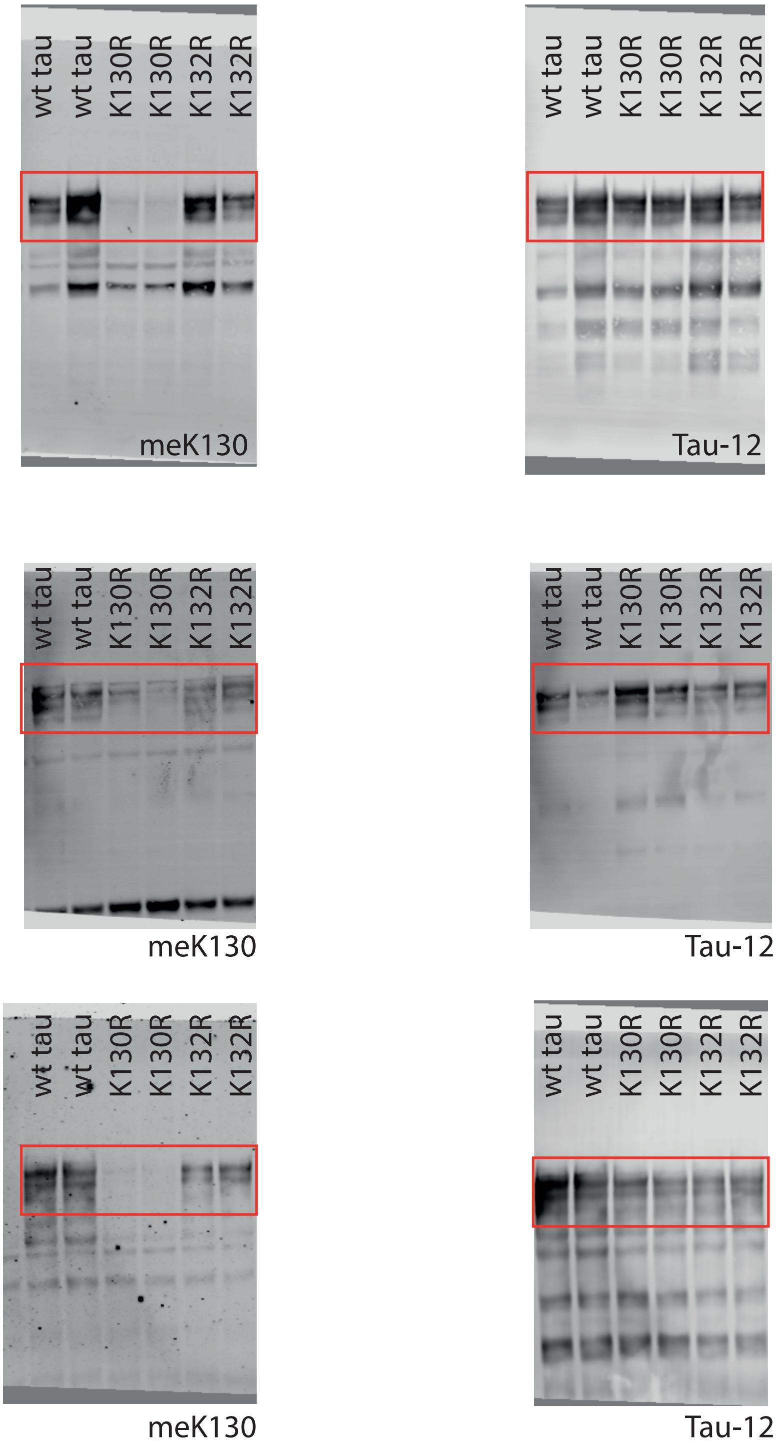

Full Western blot images related to Fig. 6D
