# Supplementary material for: SETD7-mediated monomethylation is enriched on soluble Tau in Alzheimer’s disease
Source: Mol Neurodegener. 2021 Jul 2;16:46. doi: 10.1186/s13024-021-00468-x (PMC8254302; doi:10.1186/s13024-021-00468-x)
Supplement: Supplementary file 3 — Additional file 3: Suppl Table S1. Characteristics of human brain samples used for Tau PTM discovery. Suppl. Table S2. PKMT inhibitors and concentrations used in cell culture. Suppl. Table S3. PTM modifications identified on tau protein immunoprecipitated from human entorhinal cortex. Trypsin and AspN digests of tau were analyzed by LC-MS/MS and resulting m/z data was analyzed by MASCOT using 10 ppm mass tolerance for the search. 1amino acid (aa) residue numbering conforms to human 2N4R tau, except for peptide 68-97 (pS113). 2aa residue according to human 1N4R tau. 3K modified lysine, S modified serine, T modified threonine. 4Enzyme used to generate peptides for MS. 5modified site according to human 2N4R tau. me: monomethylation, ac: acetylation, p: phosphorylation, MW: molecular weight in Dalton. Suppl. Table S4. Characteristics of human brain samples used for sarkosyl extraction. Suppl. Table S5. Subcellular localization of PKMTs identified by MS. [file 13024_2021_468_MOESM3_ESM.docx]

| Sample | Age | Sex | Braak stage | PMI [h] | Cause of death | Source |
| --- | --- | --- | --- | --- | --- | --- |
| Control 1 | 77 | M | 0 | 17.45 | Carcinomatosis, cancer of unknown primary | Bristol brain bank |
| Control 2 | 72 | M | I | 16.25 | Multiple myeloma, paroxysmal atrial fibrillation | Bristol brain bank |
| Control 3 | 96 | F | I | 72 | Urinary sepsis, broxysmal aterial fibrillation, hypertension | King's college London |
| Control 4 | 78 | M | I | 30.5 | Metastatic renal carcinoma, bilateral pulmonary emboli | King's college London |
| Control 5 | 87 | M | I | 48 | Cardiac failure | King's college London |
| Control 6 | 78 | M | 0 | 56 | Metastatic brain tumor | Bristol brain bank |
| Control 7 | 86 | F | I | 38.5 | Cerebrovascular disease | Bristol brain bank |
| Control 8 | 96 | F | I | 49 | Cerebrovascular disease | Bristol brain bank |
| Control 9 | 92 | M | I | 56,5 | Cerebral amyloid angiopathy | Bristol brain bank |
| AD 1 | 80 | M | III | 50 | Bronchopneumonia, idiopathic pulmonary fibrosis | Bristol brain bank |
| AD 2 | 81 | M | IV | 38 | Lewy body dementia | Bristol brain bank |
| AD 3 | 83 | F | III | 70.75 | Neutropenic sepsis, endometrial carcinoma, ischaemic heart disease | Bristol brain bank |
| AD 4 | 95 | F | IV | 87.5 | Frailty of old age | Bristol brain bank |
| AD 5 | 91 | F | III | 27.5 | Frailty of old age, cerebrovascular disease, atrial fibrillation | Bristol brain bank |
| AD 6 | 95 | M | III | 25.45 | Corticobasal degeneration | Bristol brain bank |
| AD 7 | 85 | F | III | 13.5 | Old age | Bristol brain bank |
| AD 8 | 95 | F | III | 65.5 | Alzheimer’s disease | Bristol brain bank |
| AD 9 | 82 | M | III | 51.25 | Alzheimer’s disease | Bristol brain bank |

**Suppl Table S1:** Characteristics of human brain samples used for Tau PTM discovery.

| compound | Supplier/cat. no. | PKMT target | stock concentration (DMSO) | Final concentration |
| --- | --- | --- | --- | --- |
| SGC0946 | Sigma SML1107 | DOT1L | 10 mM | 1 µM |
| GSK343 | Sigma SML0766 | EZH2 | 10 mM | 1 µM |
| UNC0642 | Sigma SML1037 | G9A/GLP | 10 mM | 1 µM |
| A-196 | Sigma SML1565 | KMT5B/C | 10 mM | 10 µM |
| BI-9321 | Bio-techne 6665 | NSD3 | 10 mM | 10 µM |
| BAY-598 | Sigma SML1603 | SMYD2 | 5 mM | 1 µM |
| BAY-6035 | Sigma SML2325 | SMYD3 | 5 mM | 1 µM |

**Suppl. Table S2**: **PKMT inhibitors and concentrations used in cell culture.**

| aa residues^1^ | Peptide^3^ | Enzyme^4^ | Site^5^ | PTM | MW Da | Error p.p.m | MASCOT  score |
| --- | --- | --- | --- | --- | --- | --- | --- |
| 68-97^2^ | STPTAEAEEAGIGDTPSLEDEAAGHVTQAR | Try | 113 | p | 3089.34624 | 1.14 | 51 |
| 116-132 | DEAAGHVTQARMVSKSK | AspN | 130 | me | 1827.92612 | 1.52 | 58 |
| 116-137 | DEAAGHVTQARMVSKSKDGTGS | AspN | 132 | me | 2245.07571 | -1.45 | 87 |
| 144-155 | GADGKTKIATPR | Try | 150 | me | 1227.69353 | 1.91 | 49 |
| 156-180 | GAAPPGQKGQANATRIPAKTPPAPK | Try | 163 | ac | 2465.35025 | -1.47 | 32 |
| 175-194 | TPPAPKTPPSSGEPPKSGDR | Try | 181 | p | 2081.97829 | -1.02 | 98 |
| 175-209 | TPPSSGEPPKSGDRSGYSSPGSPGTPGSR | Try | 198 | p | 2865.25667 | -0.30 | 36 |
| 195-209 | SGYSSPGSPGTPGSR | Try | 199 | p | 1472.59331 | -0.63 | 52 |
| 195-209 | SGYSSPGSPGTPGSR | Try | 202 | p | 1472.59331 | 1.53 | 72 |
| 212-224 | TPSLPTPPTREPK | Try | 217 | p | 1499.73850 | 0.67 | 32 |
| 225-240 | KVAVVRTPPKSPSSAK | Try | 231 | p | 1730.94440 | 0.29 | 54 |
| 226-240 | VAVVRTPPKSPSSAK | Try | 231/235 | p/p | 1682.81577 | -1.75 | 43 |
| 258-274 | SKIGSTENLKHQPGGGK | Try | 259 | me | 1750.93259 | -0.85 | 32 |
| 258-267 | SKIGSTENLK | Try | 262 | p | 1155.55364 | 0.57 | 55 |
| 258-274 | SKIGSTENLKHQPGGGK | Try | 263 | p | 1816.88326 | 1.41 | 53 |
| 283-294 | DLSNVQSKCGSK | AspN | 294 | me | 1335.64526 | -0.57 | 94 |
| 291-317 | CGSKDNIKHVPGGGSVQIVYKPVDLSK | Try | 298 | me | 2895.52768 | -3.30 | 39 |
| 341-353 | SEKLDFKDRVQSK | Try | 343 | me | 1592.85220 | -0.82 | 38 |
| 341-353 | SEKLDFKDRVQSK | Try | 353 | me | 1592.85220 | -1.28 | 49 |
| 384-406 | AKTDHGAEIVYKSPVVSGDTSPR | Try | 385 | me | 2427.23941 | 0.92 | 45 |
| 396-406 | SPVVSGDTSPR | Try | 403 | p | 1180.51254 | 1.35 | 55 |
| 386-406 | TDHGAEIVYKSPVVSGDTSPR | Try | 404 | p | 2294.05801 | 0.10 | 72 |
| 430-441 | DEVSASLAKQGL | AspN | 438 | me | 1230.64557 | 0.42 | 47 |

**Suppl. Table S3**: PTM modifications identified on tau protein immunoprecipitated from human entorhinal cortex. Trypsin and AspN digests of tau were analyzed by LC-MS/MS and resulting m/z data was analyzed by MASCOT using 10 ppm mass tolerance for the search. ^1^amino acid (aa) residue numbering conforms to human 2N4R tau, except for peptide 68-97 (pS113). ^2^aa residue according to human 1N4R tau. ^3^K modified lysine, S modified serine, T modified threonine. ^4^Enzyme used to generate peptides for MS. ^5^modified site according to human 2N4R tau. me: monomethylation, ac: acetylation, p: phosphorylation, MW: molecular weight in Dalton.

| Sample | Age | Sex | Braak stage | PMI [h:min] | Cause of death | Source |
| --- | --- | --- | --- | --- | --- | --- |
| 1 | 93 | M | 0 | 07:40 | Heart failure | NBB |
| 2 | 85 | F | 1 | 07:05 | Terminal renal insufficiency | NBB |
| 3 | 89 | F | 1 | 13:00 | Suicide by medication | NBB |
| 4 | 90 | M | 2 | 05:45 | Unknown | NBB |
| 5 | 88 | F | 2 | 06:52 | Euthanasia | NBB |
| 6 | 76 | F | 2 | 04:45 | Adenocarcinoma | NBB |
| 7 | 80 | F | 3 | 07:04 | Euthanasia | NBB |
| 8 | 95 | F | 3 | 07:05 | Liver abscess with sepsis | NBB |
| 9 | 89 | F | 3 | 06:35 | Heart failure and dehydration | NBB |
| 10 | 88 | F | 3 | 05:25 | Abdominal haematoma | NBB |
| 11 | 91 | F | 4 | 06:05 | Respiratory insufficiency | NBB |
| 12 | 96 | F | 4 | 07:20 | Heart failure, Aortic valve stenosis, hypertension and dementia | NBB |
| 13 | 82 | F | 4 | 05:55 | Cardiac arrest with dehydration after myocardial infarction | NBB |
| 14 | 81 | F | 4 | 06:15 | General physical deterioration by collum fracture with complications and AD | NBB |
| 15 | 91 | M | 4 | 04:10 | Acute Myeloid Leukemia and Pneumonia | NBB |
| 16 | 73 | F | 5 | 04:45 | Sepsis/cachexia | NBB |
| 17 | 82 | M | 5 | 05:15 | Cachexia due to swallowing problems caused by advanced dementia syndrome | NBB |
| 18 | 78 | M | 5 | 06:35 | Fever of unknown origin, cachexia and dehydration by dementia syndrome | NBB |
| 19 | 84 | F | 6 | 04:05 | Heart failure | NBB |
| 20 | 64 | M | 6 | 04:35 | Palliative sedation for untreatable pains by kyphosis | NBB |
| 21 | 82 | F | 6 | 05:00 | Cachexia/dehydration | NBB |

**Suppl. Table S4:** Characteristics of human brain samples used for sarkosyl extraction.

| PKMT | cytoplasmic | nuclear | chromatin-bound |
| --- | --- | --- | --- |
| DOT1L |  |  | x |
| EHMT1 |  | x | x |
| EHMT2 |  | x | x |
| EZH1 | x | x |  |
| EZH2 |  | x | x |
| KMT2A |  | x | x |
| KMT2B |  | x |  |
| KMT2C |  | x |  |
| KMT2D |  | x |  |
| KMT2E |  | x |  |
| NSD1 |  | x |  |
| PRDM16 |  | x |  |
| PRDM2 |  | x | x |
| SETD1A |  | x | x |
| SETD1B | x | x |  |
| SETD2 |  | x | x |
| SETD7 | x |  |  |
| SETDB1 | x | x |  |
| SMYD3 | x | x |  |
| SUV39H2 |  |  | x |

**Suppl. Table S5:** Subcellular localization of PKMTs identified by MS.
